# Potential Hybridization of *Fasciola hepatica* and *F. gigantica* in Africa—A Scoping Review

**DOI:** 10.3390/pathogens11111303

**Published:** 2022-11-06

**Authors:** Sophy Nukeri, Mokgadi Pulane Malatji, Mita Eva Sengupta, Birgitte Jyding Vennervald, Anna-Sofie Stensgaard, Mamohale Chaisi, Samson Mukaratirwa

**Affiliations:** 1 School of Life Science, College of Agriculture, Engineering and Science, University of KwaZulu-Natal, Westville Campus, Durban 4001, South Africa; 2 Foundational Research & Services, South African National Biodiversity Institute, Pretoria 0001, South Africa; 3 Department of Veterinary and Animal Sciences, Faculty of Health and Medical Sciences, University of Copenhagen, 1870 Copenhagen, Denmark; 4 Center for Macroecology, Evolution and Climate Change, Globe Institute, Faculty of Health and Medical Sciences, University of Copenhagen, 1870 Copenhagen, Denmark; 5 Department of Veterinary Tropical Diseases, University of Pretoria, Onderstepoort 0110, South Africa; 6 One Health Center for Zoonoses and Tropical Veterinary Medicine, Ross University School of Veterinary Medicine, Basseterre KN 0101, Saint Kitts and Nevis

**Keywords:** *Fasciola hepatica*, *F. gigantica*, hybrids, parthenogenetic species, distribution, snail intermediate host, Africa

## Abstract

The occurrence of *Fasciola gigantica* and *F. hepatica* in Africa is well documented; however, unlike in Asia, there is a paucity of information on the existence of hybrids or parthenogenetic species on the continent. Nonetheless, these hybrid species may have beneficial characteristics, such as increased host range and pathogenicity. This study provides evidence of the potential existence of *Fasciola* hybrids in Africa. A literature search of articles published between 1980 and 2022 was conducted in PubMed, Google Scholar, and Science Direct using a combination of search terms and Boolean operators. *Fasciola* species were documented in 26 African countries with *F. hepatica* being restricted to 12 countries, whilst *F. gigantica* occurred in 24 countries, identified based on morphological features of adult *Fasciola* specimens or eggs and molecular techniques. The co-occurrence of both species was reported in 11 countries. However, the occurrence of potential *Fasciola* hybrids was only confirmed in Egypt and Chad but is suspected in South Africa and Zimbabwe. These were identified based on liver fluke morphometrics, assessment of the sperms in the seminal vesicle, and molecular techniques. The occurrence of intermediate host snails *Galba truncatula* and *Radix natalensis* was reported in Ethiopia, Egypt, South Africa, Tanzania, and Uganda, where *F. hepatica* and *F. gigantica* co-occurrences were reported. The invasive *Pseudosuccinea columella* snails naturally infected with *F. gigantica* were documented in South Africa and Egypt. In Zimbabwe, *P. columella* was infected with a presumed parthenogenetic *Fasciola*. This suggests that the invasive species might also be contributing to the overlapping distributions of the two *Fasciola* species since it can transmit both species. Notwithstanding the limited studies in Africa, the potential existence of *Fasciola* hybrids in Africa is real and might mimic scenarios in Asia, where parthenogenetic *Fasciola* exist in most Asian countries. In South Africa, aspermic *F. hepatica* and *Fasciola* sp. have been reported already, and *Fasciola* hybrids have been reported? in Chad and Egypt. Thus, the authors recommend future surveys using molecular markers recommended to identify *Fasciola* spp. and their snail intermediate hosts to demarcate areas of overlapping distribution where *Fasciola* hybrids and/or parthenogenetic *Fasciola* may occur. Further studies should also be conducted to determine the presence and role of *P. columella* in the transmission of *Fasciola* spp. in these geographical overlaps to help prevent parasite spillbacks.

## 1. Introduction

Fasciolosis is an important food and water-borne zoonotic infection of human, domestic, and wild animals mainly caused by the two liver flukes, *Fasciola hepatica* (Linnaeus, 1758) and *F. gigantica* (Cobbold, 1856) [1,2,3]. Of the two species, *F. hepatica* has been shown to have a cosmopolitan distribution occurring in five continents except for Antarctica [4,5], whilst *F. gigantica* is restricted to the tropical and subtropical regions of Asia and Africa [6]. According to [7], the distribution of both *Fasciola* species is associated with the availability and dispersal of viable snail vectors that act as - intermediate hosts (IHs) as well as climatic and ecological conditions suitable for the survival of these snails [8,9,10].

Previous data have suggested that *F. hepatica* originated from Eurasian ovicaprines, especially *Ovis* species [11]. This concept has been generally accepted due to the dispersion of its preferred IH snail, *Galba truncatula* (Müller, 1774), which is associated with areas with mild and cold climates [12], and very high altitudes [11]. Recent data elucidated that the ancestral fasciolids of *F. hepatica* and *F. gigantica* are speculated to have emerged in the lowlands of East Africa [13], followed by speciation of *F. hepatica* in the Eurasian Near East and *F. gigantica* in Africa [11]. *Fasciola hepatica* then spread from Eurasia to other parts of the world [11] where its main IH is *G. truncatula* [14,15]. In Africa, the cryptic species *Galba mweruensis* (Connolly, 1929) has been indicated as an IH of both *Fasciola* species [16,17] but has only been proven to transmit *F. hepatica* in Lesotho and Ethiopia [18,19].

Based on [11], the assumption that the emergence of Fasciolinae species and secondary colonization by *F. gigantica* and other *Fasciola* species in Africa was favored by a switch of IHs from planorbid to lymnaeid snails as stated by [20] does not fit the current knowledge. *Fasciola gigantica* is distributed throughout western, sub-Saharan, and eastern Africa following the wide distribution of the snail intermediate host species *Radix natalensis* (Krauss, 1848) that it adapted to [11]. Furthermore, this fasciolid species has adapted to ruminants from families Giraffidae, Reduncinae, and Alcelaphinae in sub-Saharan Africa as definitive hosts [11]. Following the movement of animals facilitated by humans and the subsequent introduction of intermediate host snail species into new areas, *F. gigantica* spread to other regions. In these regions, its main IHs are the *Radix* species of the “auricularia super-species” (Hubendick, 1951) *R. rubiginosa* (Minchelin, 1831) in Asia, and *R. natalensis* in Africa [9,21].

The spread of both *Fasciola* species led to overlapping distributions in areas where climatic conditions allow the IHs of both species to survive and co-exist, particularly in tropical regions of Africa and Asia [11,22]. In South Africa, the invasive snail *Pseudosuccinea columella* (Say, 1817) has been suspected to be the vector snail responsible for possibly transmitting both *Fasciola* species, thus contributing towards the overlap [23], following an observed increase in infection rates of both *Fasciola* species coinciding with the introduction of the invasive snail species in the country [24]. *Pseudosuccinea columella* has been reported to be responsible for the secondary transmission of *F. hepatica* in South America and the Caribbean region [9,25], and it has been proven to naturally transmit *F. gigantica* in Africa [23,26]. Therefore, it can be hypothesized that *P. columella* may potentially be transmitting *F. hepatica* in Africa, supported by successful experimental infections of the Egyptian *P. columella* population with *F. hepatica* [27] and natural infections with an unknown yet suspected *Fasciola* hybrid in South Africa [23] and Zimbabwe [28], thus facilitating the overlapping geographical distribution of *Fasciola* species in some African countries as reported in Egypt [29] and South Africa [30].

In areas of geographical overlap, hybridization between *F. hepatica* and *F. gigantica* has been reported, resulting in *Fasciola* hybrid species consisting of admixed/introgressive genotypes due to interspecific mating [31,32]. According to [13], this hybridization may have occurred about 2000 years ago in China, resulting in parthenogenetic populations which were then spread across multiple Asian countries where they co-exist with *F. gigantica*. However, due to a lack of standardized methods for identifying these populations, the parthenogenetic *Fasciola* in Japanese populations was initially misidentified as *F. hepatica* [33]. Moreover, parthenogenetic *Fasciola* was previously called aspermic *Fasciola* but the discovery of few aspermic triploid individuals that produced and stored mature sperms in their seminal vesicles led to this term being disregarded. Thus, parthenogenetic *Fasciola* can be distinguished from other *Fasciola* hybrids by its ability to reproduce and maintain successive generations [13].

Although [13] suggests that the parthenogenetic *Fasciola* populations have not been reported outside of Asia, other forms of *Fasciola* hybrid populations have been suspected in some African countries, as evidenced by the recent reports of aspermic *F. hepatica* and unidentified *Fasciola* sp. [23,28,30]. However, they could have been missed outside Asia due to the paucity of research on the occurrence of *Fasciola* hybrids in the areas of geographical overlap [30,34]. Moreover, African studies have not applied nuclear phosphoenolpyruvate carboxykinase (PEPCK) and DNA polymerase delta (POLD) markers which are recommended as suitable markers to identify parthenogenetic *Fasciola* [13]. Nonetheless, knowledge of the occurrence of *Fasciola* hybrids is significant since hybrid forms may have increased in their geographic and host expansion [2]. Hence, this review aimed to assess the possible hybridization of *F. hepatica* and *F. gigantica* resulting in parthenogenetic *Fasciola* in Africa as presented in published literature, and the role of *P. columella* in driving the convergence of the two *Fasciola* spp., allowing hybridization in the process.

## 2. Materials and Methods

The scoping review was conceptualized to address the following review questions: (1) What is the distribution of *Fasciola* species in Africa? (2) Which African countries have co-occurrences/overlapping geographical distribution of *F. hepatica* and *F. gigantica*? (3) Which intermediate snail hosts are implicated in these overlapping distributions? (4) Has the hybridization of *F. hepatica* and *F. gigantica* occurred in Africa? (5) If no, are there signs that hybridization is in the process or potentially occurring in Africa but under-documented? (6) If yes, is it occurring in the same way as in China, where two pure parental *Fasciola* species are hybridizing or other Asian countries where pure maternal *F. gigantica* is hybridizing with parthenogenetic species? To answer these questions, articles published in peer-reviewed journals reporting on the distribution of *F. hepatica*, *F. gigantica*, snail IHs, and the occurrence of *Fasciola* hybrids/parthenogenetic *Fasciola* populations in Africa were retrieved and appraised. Guidelines from the Preferred Reporting Items for Systematic Reviews and Meta-Analyses (PRISMA) were followed using the approaches for scoping review described by [35].

### 2.1. Search Strategy

A literature search was conducted on Google Scholar, PubMed, and Science Direct electronic databases. The search was completed using a combination of Boolean operators (OR, AND) and the following search terms: *F. hepatica* AND *F. gigantica* AND co-occurrence, *Fasciola* spp. AND intermediate hosts, *Fasciola* species AND hybridization, *Fasciola* hybrids OR Intermediate forms of *Fasciola* OR Parthenogenetic *Fasciola* in Africa (Algeria OR Angola OR Benin OR Botswana OR Burkina Faso OR Burundi OR Cameroon OR Cape Verde OR Central African Republic OR Chad OR Comoros OR Congo OR Côte d’Ivoire OR Djibouti OR DR Congo OR Egypt OR Equatorial Guinea OR Eritrea OR Ethiopia OR Gabon OR The Gambia OR Ghana OR Guinea OR Guinea-Bissau OR Kenya OR Lesotho OR Liberia OR Libya OR Madagascar OR Malawi OR Mali OR Mauritania OR Mauritius OR Morocco OR Mozambique OR Namibia OR Niger OR Nigeria OR Réunion OR Rwanda OR Sao Tome and Principe OR Senegal OR Seychelles OR Sierra Leone OR Somalia OR South Africa OR South Sudan OR Sudan OR Swaziland OR Tanzania OR Togo OR Tunisia OR Uganda OR Western Sahara OR Zambia OR Zimbabwe). Articles with relevant information were identified by screening titles and abstracts to obtain relevant articles. Full texts of relevant articles were retrieved and managed using EndNote reference manager version X9 (Clarivate Analytics, Philadelphia, PA, USA).

### 2.2. Inclusion and Exclusion Criteria

Articles were included in the study if they were published in peer-reviewed journals and they: (1) precisely reported on the occurrences and co-occurrences of *F. hepatica* and *F. gigantica* and their snail intermediate hosts in Africa; (2) reported on the occurrences of *Fasciola* hybrids or parthenogenetic *Fasciola* in Africa; (3) identified eggs or adult flukes up to species level using molecular techniques or morphological features; and (4) were conducted in Africa and published between January 1980 and February 2022.

The review excluded articles that (1) did not identify *Fasciola* species and their intermediate hosts up to species level; (2) reported on experimental infections; (3) did not contribute towards answering the research questions; and (4) were not conducted in Africa or were published outside the years indicated above and were not written in English.

### 2.3. Charting, Collating, and Summarising Data

Data were extracted from articles that met the inclusion criteria after appraisal. Data concerning the details of the authors and year of publication, the aim or objectives of the study, the country where the study was conducted, the outcomes of the study, and any information relevant to the main objectives of this review were extracted and recorded.

## 3. Results

**Figure 1:**
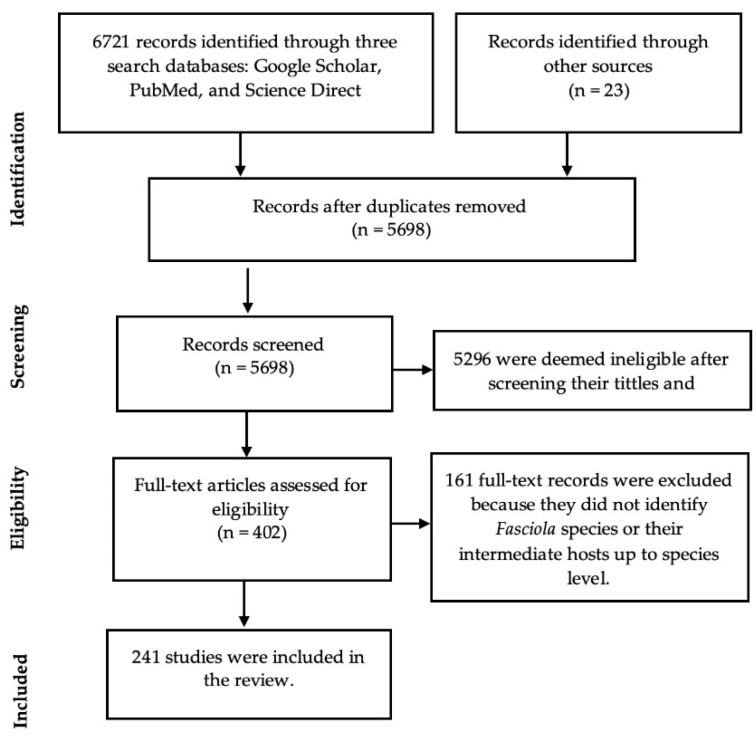
PRISMA diagram.

The searches using Google Scholar, PubMed, and Science Direct electronic databases yielded 6721 records, and 23 additional records were identified through reference list screening (Figure 1). A total of 1023 duplicates were identified and removed. The titles and abstracts of 5698 records were screened for relevance and 5296 records were deemed irrelevant and excluded. Full-text articles of 402 records were extracted and assessed for eligibility, and 161 records were deemed ineligible and excluded because they did not contribute towards answering the review questions. A total of 241 records from 26 African countries were included in the scoping review (Table S1 and Table 2).

Results showed that *Fasciola* spp. were predominantly reported from cattle (*Bos taurus*) (Linnaeus, 1758) (n = 159), and also from other vertebrate hosts including sheep (*Ovis aries*) (Linnaeus, 1758) (n = 42), goat (*Capra hircus*) (Linnaeus, 1758) (n = 27), African buffalo (*Syncerus caffer*) (Sparrman, 1779) (n = 9), donkey (*Equus africanus*) (Linnaeus, 1758) (n = 5), horse (*Equus ferus*) (Linnaeus, 1758) (n = 3), humans (*Homo sapiens*) (Linnaeus, 1758) (n = 3), antelope (*Hippotragus niger*) (Harris, 1838) (n = 2), pig (*Sus domesticus*) (Erxleben, 1777) (n = 2), mule (*Equus mulus*) (n = 1), camel (*Camelus dromedarius*) (Linnaeus, 1758) (n = 1), eland (*Taurotragus oryx*) (Pallas, 1766) (n = 1), duiker (*Sylvicapra grimmia*) (Linnaeus, 1758 (n = 1), impala (*Aepyceros melampus*) (Lichtenstein, 1812) (n = 1), Kafue lechwe (*Kobus leche*) (Gray, 1850) (n = 1), and kudu (*Tragelaphus strepsiceros*) (Pallas, 1766) (n = 1).

**Figure 2:**
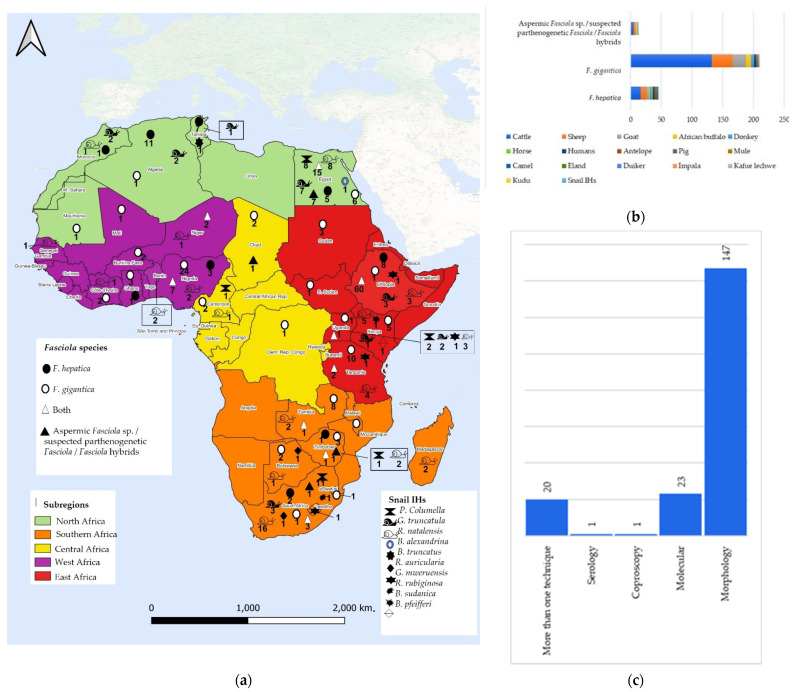
(**a**) A map showing the geographical distribution and occurrence of *Fasciola* spp. and their snail intermediate hosts in Africa based on records retrieved in the scoping review. The taxa reported are symbolized next to the number of studies in each country; (**b**) Reviewed studies that reported on *Fasciola* infection in various definitive hosts; (**c**) Techniques used in reviewed studies to identify *Fasciola* spp.

Most studies identified *Fasciola* specimens based on morphological features (n = 147), whilst 23 (n = 23) studies identified the parasites using molecular techniques. Other studies used egg morphology from coproscopy (n = 1), serology (n = 1), and 20 (n = 20) studies used a combination of more than one diagnostic technique (Table S1 and Figure 2).

### 3.1. Geographical Distribution and Occurrence of F. hepatica and F. gigantica in Africa

Results show that *Fasciola* species occur in all five African subregions (Figure 2). In North Africa, both *F. hepatica* and *F. gigantica* have been reported in Egypt and Algeria, whilst Morocco and Tunisia recorded the occurrence of *F. hepatica* only and *F. gigantica* was recorded in Sudan and South Sudan. In East Africa, both *Fasciola* species have been reported in Ethiopia, Uganda, and Tanzania, whilst Kenya and Malawi reported the occurrence of *F. gigantica* only. Reviewed studies further revealed that *F. gigantica* was the only fasciolid documented in Central African countries, viz., Cameroon, Chad, and the Democratic Republic of Congo. A total of five Southern African countries reported the occurrence of *Fasciola* species with Swaziland and Botswana recording *F. gigantica* only, whereas South Africa, Zambia, and Zimbabwe reported both *F. gigantica* and *F. hepatica*. In West Africa, Burkina Faso, Côte d’Ivoire, Mali, and Mauritania recorded the occurrence of only *F. gigantica,* whilst Ghana, Niger, and Nigeria recorded both *Fasciola* species (Table S2).

### 3.2. Checklist of Snail Intermediate Hosts of F. hepatica and F. gigantica in Africa

Evidence from the reviewed studies shows that both *G. trancatula* and *R. natalensis* snails occur in North, South, and East African subregions, whilst West and Central regions only documented the occurrence of *R. natalensis* (Table 1). Additionally [16], documented the presence of a cryptic *Galba* species, *G. mweruensis*, in Southern Africa (Lesotho) and East Africa (Ethiopia, Uganda, and Tanzania). In North Africa, both *R. natalensis* and *G. truncatula* were documented specifically in Egypt, whilst Algeria, Morocco, and Cameroon documented the presence of *G. truncatula* only. Reviewed studies further showed that *Fasciola* species in North Africa can infect other snail species with evidence of natural infections in *Biomphalaria alexandrina* (Ehrenberg, 1831) and *P. columella* by Egyptian *F. gigantica* and *Bulinus truncatus* (Audouin, 1827) by Tunisian *F. hepatica* (Table S3). In Central Africa, *R. natalensis* and *P. columella* were the only lymnaeid species documented in Cameroon. Southern African region showed the highest species diversity by documenting five lymnaeid species including *P. columella*, *R. natalensis, R. auricularia* (Linnaeus, 1758), *R. rubiginosa* (Michelin, 1831) and *G. truncatula*. South Africa documented all five lymnaeid species whilst Zimbabwe and Namibia recorded *R. natalensis* and *P. columella*. Zambia and Lesotho recorded *R. natalensis* and *G. truncatula*, respectively. Botswana recorded *R. natalensis* and *R. auricularia*. Evidence of natural infections in *P. columella* with *Fasciola* sp. was documented in Zimbabwe and South Africa. In West Africa, *R. natalensis* was the only lymnaeid species documented in Benin, Côte d’Ivoire, Niger, Nigeria, and Senegal. East African countries where *R. natalensis* and *G. truncatula* were documented include Ethiopia, Tanzania, and Uganda, whilst Kenya and Madagascar reported *R. natalensis* (Table 1). Results further showed evidence of infection of *Bio. pfeifferi* (Krauss, 1848) and *Bio. sudanica* (Martens, 1870) by *F. gigantica* in Kenya (Table S3).

### 3.3. Occurrence and Identification of Fasciola Hybrids/Aspermic and Suspected Parthenogenetic Fasciola in Africa

Results show that *Fasciola* hybrids were confirmed in cattle from Egypt and Chad, and in buffalo and sheep from Egypt. These *Fasciola* hybrids were genetically identified by comparing the nucleotides of the ITS-1 and ITS-2 sequences and individuals displayed heterozygosity with the ITS-Fh/Fg genotype thus confirming mixed bases of both *Fasciola* types at the variable sites [1,2,29]. Furthermore, studies also compared the nuclear ITS gene sequences to the mitochondrial sequences of NADH dehydrogenase subunit I (NDI) and cytochrome c-oxidase subunit I (COI) regions. The hybrid individuals identified as one *Fasciola* species at ITS-1 and ITS-2, however, displayed sequences of the other species at NDI and/or COI (Table 2). Morphologically, the length/width ratio of *Fasciola* hybrid adult flukes (1.86–3.37 mm) significantly differed from those of *F. gigantica* (3.43–5.50 mm) and *F. hepatica* (1.65–2.76 mm) (Table 2). Hybrid species also showed variations in the size and position of the oral and ventral suckers, the structure of intestinal caeca, and the position and branches of testes from *F. hepatica* and *F. gigantica* (Table 2). Results also showed that some specimens which possessed morphometric characters of *F. hepatica* displayed a close genetical relation to *F. gigantica* based on the PCR-linked restriction fragment length polymorphism (PCR-RFLP) using the AvaII restriction enzymes [105].

Parthenogenetic *Fasciola* were suspected in the form of aspermic *Fasciola* species from cattle in South Africa. The aspermic *Fasciola* sp. (suspected parthenogenetic) were characterized as specimens with no sperms in their seminal vesicles or had “very scanty sperms” and mean length/width ratio measurements that significantly varied from those of *F. gigantica* and *F. hepatica* which were 2.02 ± 0.35 mm, 2.79 ± 0.48 mm and 4.41 ± 1.10 mm, respectively [30]. Unidentified *Fasciola* sp. isolated from *P. columella* were recorded in Zimbabwe and South Africa (Table S2 and Table 2). In Zimbabwe, the unidentified *Fasciola* sp. showed a close affinity to *F. gigantica* and *F. hepatica* on a phylogenetic tree based on the ITS marker [28]. In South Africa, the unidentified *Fasciola* sp. Showed a close affinity to *F. gigantica* on BLAST and genetic distance; however, they formed their own haplotype and clade different from that of *F. gigantica* and *F. hepatica* based on the ITS-1 marker [23].

## 4. Discussion

Previous studies have indicated that *F. hepatica* has a cosmopolitan distribution [4,5], whereas *F. gigantica* is restricted to parts of Asia and Africa [6]. Reviewed studies have confirmed that *F. gigantica* is more widespread in Africa as expected following the distribution of its IHs [100] and was reported in 24 African countries. In contrast, *F. hepatica* was more restricted in its distribution, corresponding to the restricted distribution of its IHs which occur in cooler parts of Africa [100]. *Fasciola hepatica* was reported in a few countries including Egypt and the Maghreb countries (Algeria, Morocco, and Tunisia) in North Africa; South Africa, Zimbabwe, and Zambia in Southern Africa; Nigeria and Niger in West Africa; and Tanzania, Uganda, and Ethiopia in East Africa, which corresponded to reports from previous studies [22,110,111,112]. The results also showed co-occurrences of the two *Fasciola* species in Algeria, Ghana, Ethiopia, Egypt, Nigeria, South Africa, Niger, Tanzania, Uganda, Zambia, and Zimbabwe.

*Fasciola* specimens were predominately collected from their “primary domestic definitive hosts” [113], i.e., cattle, sheep, and goats in all subregions. This is not surprising since these mammalian hosts easily consume *Fasciola* metacercariae from pastures [114] when grazing in areas near water bodies which are habitats of the snail IHs [115]. Results also showed that natural infections of *F. hepatica* in Africa occurred in cattle, African buffalo, sheep, goat, camel, humans, pig, horse, antelope, duiker, kudu, mule, and donkey, whilst *F. gigantica* infected cattle, sheep, goat, impala, Kafue lechwe, donkey, African buffalo, humans, antelope, horse, duiker, mule, and eland (Figure 2). The low number of studies reporting fasciolosis in wildlife supports a suggestion by [116,117,118] that infections might be accidental and a result of shared drinking water between wildlife and cattle since most wildlife animals are browsers and thus less likely to become infected through aquatic vegetation [22]. Moreover, a few reviewed studies reported infections in humans, thus highlighting that human fasciolosis is either occurring at a very low rate or neglected since humans can easily become infected by ingesting watercress or other edible raw plants contaminated with metacercariae, which form part of the regular diet in several countries [119,120,121], or through drinking water contaminated with metacercariae. Studies from North, East, and Southern Africa recorded infections in buffalo, donkey, horse, mule, camel, and pig. A few South, West, and East African studies reported infections in humans and wild animals including antelope, eland, duiker, impala, Kafue lechwe, and kudu.

Reviewed studies show that both *G. truncatula* and *R. natalensis* co-occur in Ethiopia, Egypt, South Africa, Tanzania, and Uganda, and these are the countries where both *F. hepatica* and *F. gigantica* have been documented. However, other countries such as Algeria, Ghana, Nigeria, Niger, Zambia, and Zimbabwe reported co-occurrence of both *F. hepatica* and *F. gigantica,* with only the presence of one IH being documented. According to [111], such outcomes can be attributable to livestock transhumance, or these species use other freshwater snails from other families and not Lymnaeidae or Racidinae and we further add that limited or no effort has been made to look for the IH. However, altitude and topography have been reported to have an influence on the survival of the snail IHs, thus contributing to the occurrence of *Fasciola* species [22,100,103]. No records on the occurrence of snail IHs of *Fasciola* species were retrieved for nine African countries (South Sudan, Sudan, Chad, DR Congo, Burkina Faso, Ghana, Mali, Mauritania, and Malawi), thus highlighting the paucity of research on snail IHs-*Fasciola* spp. in these African countries. Moreover, the presence of lymnaeid snail IHs were reported in Lesotho, Namibia, Benin, Senegal, and Madagascar, where there was no reviewed evidence of *Fasciola* spp. This raises concern about possible rapid transmission should the trematodes be introduced, and according to [122] it might be harmful to inhabitants should there be an introduction of the parasite by infected livestock near water bodies with IHs and accessed by humans during their anthropogenic activities.

Reviewed studies showed that apart from *G. truncatula*, *F. hepatica* has been observed to naturally infect *B. truncatus* in Tunisia [51]. Additionally, *G. mweruensis*, based on studies by [16], appears to be well established as a major IH of *F. hepatica* throughout the Sub-Saharan Africa region and has been reported in Lesotho, Ethiopia, Uganda, and Tanzania. This species is presumed to be the most predominant snail species in the highlands of Ethiopia and Lesotho [18,19] and is believed to be the main IH of both *F. hepatica* and *F. gigantica* [17], and potentially other trematode infections [18,19]. Natural infections with *F. gigantica* were also detected in *Bio. alexandrina* in Egypt based on molecular techniques [123], and *Bio. Pfeifferi* and *Bio. Sudanica* in Kenya [96], and *P. columella* in South Africa [23] and Egypt [26]. In South Africa [23] and Zimbabwe [28], *P. columella* has been found naturally infected with a suspected parthenogenetic *Fasciola* sp. Considering that this invasive snail species transmit *F. hepatica* in other parts of the world, it can be suggested that *P. columella* may be responsible for the overlapping distribution of both *F. hepatica* and *F. gigantica* and, hence, promote the occurrence of *Fasciola* hybrids playing a role in the overlap between the two species.

In Asia, the occurrence of *Fasciola* hybrids has been extensively studied and documented in China [123,124], Vietnam [125,126,127,128], Japan [129,130], Korea [131,132], Bangladesh [133], Nepal [134], and Myanmar [135]. A recent study by [12] highlighted the existence of a parthenogenetic *Fasciola* population originating from hybridization between “pure” *F. gigantica* and *F. hepatica* in China at least 2000 years ago, and these populations have spread to other Asian countries including Vietnam, India, the Philipines, Thailand, Myanmar, Bangladesh, and Nepal where it co-exists with *F. gigantica*, and these have not been reported to occur outside Asia. Results from this review highlighted that hybridization may have already occurred or is in the process in some African countries including Chad, Egypt, and South Africa. This is supported by the individual flukes which have been identified to have intermediate morphological characters of *F. gigantica* and *F. hepatica* in Egypt [1,105,109] and South Africa [30], and those presumed to be *F. hepatica* with little to no sperm in their seminal vesicle in South Africa [30]. Considering that the Asian parthenogenetic *Fasciola* which were aspermic and those identified as *F. hepatica* in Japan were later classified as parthenogenetic hybrids [13], it is possible that the “aspermic” populations found in South Africa might also be parthenogenetic *Fasciola*. Thus, the presence of parthenogenetic *Fasciola* in Africa could have been missed due to a lack of specific studies focusing on the detection of hybrid populations, or the use of inappropriate or non-specific markers to identify these populations, especially in areas with overlapping distributions of these two *Fasciola* species. Itagaki [13] recommended the use of a multiplex PCR based on the PEPCK and POLD markers, but recent studies have suggested that the fatty acid binding protein type I gene markers are more reliable compared to the PEPCK and POLD markers [136].

*Fasciola* hybrids from Egypt [105] that had morphometric characters of *F. hepatica* were genetically more related to *F. gigantica*. The results also showed that similar to other countries where *Fasciola* hybrids/parthenogenetic populations were reported, these specimens were also characterized by either the ITS-Fh/Fg mixed genotype or the possession of sequences of one *Fasciola* species at ITS-1 and ITS-2 but sequences of the other species at NDI and/or COI [1,2,29,106,108]. Furthermore, the populations found in *P. columella* in South Africa [23] and Zimbabwe [28] were shown to be genetically closer to *F. gigantica* but formed their own clade on the phylogenetic tree based on the ITS-1 marker (genetic distances). Similar observations were reported by [13] that the Asian parthenogenetic *Fasciola* were genetically closer to *F. gigantica* than *F. hepatica* [137,138,139].

## 5. Conclusions

*Fasciola gigantica* and *F. hepatica* co-occur in Algeria, Ghana, Ethiopia, Egypt, Nigeria, South Africa, Niger, Tanzania, Uganda, Zambia, and Zimbabwe. However, the presence of *Fasciola* hybrids has only been confirmed in Egypt and Chad, and parthenogenetic populations are suspected in Zimbabwe and South Africa. *Galba truncatula* and *R. natalensis* are the main IH of *F. hepatica* and *F. gigantica,* respectively, in Africa. However, a cryptic *Galba* sp. (*G. mweruensis*) in Sub-Saharan Africa is worth investigating as it is claimed to be the major host of *F. hepatica* and might also be sustaining both *Fasciola* species in the continent. The role of *P. columella* in the transmission of *F. gigantica*, *F. hepatica,* and *Fasciola* sp. is not clear, and the current results suggest that the snail species might be responsible for *Fasciola* species overlaps as it was proven to transmit both *F. hepatica* and *F. gigantica* in other countries. Hence, the authors recommend that surveys should be conducted to assess if this is also the case in Africa and to detect hybrids in areas of geographical overlap using appropriate molecular and morphological techniques. Evidence suggests that *Fasciola* hybrids recorded in some African countries might be parthenogenetic *Fasciola* as reported in Asia. This calls for reliable molecular studies such as the concurrent use of ITS-1 and ITS-2 markers with the PEPCK, POLD, and ND1 markers combined with sperm observations for validation and identification of African *Fasciola* species to avoid misidentifications.

## Figures and Tables

**Table 1:** Checklist and distribution of snail intermediate hosts of *Fasciola* species reported in Africa based on studies conducted from 1980–2022.

Subregion	Country	Intermediate Host Species Recorded	References
North Africa	Algeria	*G. truncatula*	[36,37]
	Egypt	*P. columella, R. natalensis, G. truncatula*, *Bio. alexandrina*	[26,38,39,40,41,42,43,44,45,46,47,48,49]
	Morocco	*G. truncatula*	[16,50]
	Tunisia	*G. truncatula, Bulinus truncatus*	[51,52,53]
Central Africa	Cameroon	*R. natalensis*, *P. columella*	[54]
Southern Africa	Botswana	*R. auricularia*, *R. natalensis*	[55,56]
	Lesotho	*G. truncatula*, *G. mweruensis*	[16,57]
	Namibia	*R. natalensis, P. columella*	[58]
	South Africa	*P. columella*, *R. natalensis, R. auricularia*, *G. truncatula*, *R. rubiginosa*	[16,23,55,57,59,60,61,62,63,64,65,66,67,68,69,70,71,72,73,74,75,76]
	Zambia	*R. natalensis*	[77,78]
	Zimbabwe	*R. natalensis*, *P. columella*	[28,79,80,81]
West Africa	Benin	*R. natalensis*	[82,83]
	Côte d’Ivoire	*R. natalensis*	[84]
	Niger	*R. natalensis*	[85]
	Nigeria	*R. natalensis*	[86,87]
	Senegal	*R. natalensis*	[88]
East Africa	Ethiopia	*R. natalensis, G. truncatula*, *G. mweruensis*	[16,89,90,91,92]
	Madagascar	*R. natalensis*	[93,94]
	Kenya	*R. natalensis*, *Bio. pfeifferi, Bio. sudanica*	[16,95,96,97,98,99]
	Uganda	*R. natalensis, G. truncatula*, *G. mweruensis*	[16,100,101]
	Tanzania	*R. natalensis*, *Bio. pfeifferi, G. truncatula*, *G. mweruensis*	[16,98,102,103,104]

**Table 2:** Summary of studies reporting on the occurrence of *Fasciola* hybrids/intermediate forms and suspected parthenogenetic forms in Africa based on morphological and molecular techniques.

Author	Aim/Objective	Country	Host	No. of Specimen	Diagnostic Technique	Characteristics
[1]	To identify the phenotypic features and genetic characterization of adult fasciolids infecting buffaloes that were studied in Aswan, Egypt.	Egypt	Sheep	3	Morphology and molecular	- Intermediate *Fasciola* species had Body length (BL), Cone length (CL), Cone width (CW), ventral sucker diameter, and Pharynx width (PhW) measurements that overlapped between those of *F. hepatica* and *F. gigantica*. - Nucleotide bases varied from those of *F. gigantica* and *F. hepatica* at variable positions 1 and 3.
[2]	To molecularly characterize *Fasciola* flukes using the ITS-1 and 2 nuclear markers to confirm species and any hybrid forms.	Chad	Cattle	1	Molecular	- *Fasciola* hybrid showed heterozygosity at all variable sites. - Cloning and sequencing of both alleles confirmed the presence of one allele each for *F. hepatica* and *F. gigantica*.
[23]	Confirming whether *P. columella* was transmitting *F. gigantica* and/or *F. hepatica* in selected locations of KwaZulu-Natal and Eastern Cape provinces of South Africa.	South Africa	Snail IHs	1	Molecular	- *Pseudosuccinea columella* was found infected with *F. gigantica*, *Fasciola* sp., and *Echinostoma* sp. - *Fasciola* sp. showed a close affinity with *F. gigantica* on BLAST and genetic distance but formed its own haplotype and clade different from *F. gigantica* and *F. hepatica* based on the ITS-1 marker.
[28]	Assessed the prevalence of *Fasciola* sp. infections in the gastropod populations.	Zimbabwe	Snail IHs	3	Molecular	- Phylogenetic analyses showed a close affinity between suspected *Fasciola* sp. with *F. hepatica* and *F. gigantica* based on the ITS marker.
[29]	Molecularly ascertain the nature of *Fasciola* population derived from different hosts and different geographic locations in Egypt.	Egypt	Buffalo	2	Molecular	- Intermediate *Fasciola* forms had the ITS-2-Fh/Fg with mixed bases of both *Fasciola* types at all variable sites and nucleotide peaks of *F. hepatica* and *F. gigantica* overlapping at the 6 variable sites. - One isolate proved *F. gigantica* lineage at both NDI and COI markers, while the other worm was identified as *F. hepatica*.
[30]	Morphological and molecular characterization of *Fasciola* spp. collected from cattle slaughtered at abattoirs located in the two provinces of South Africa, where two species are endemic.	South Africa	Cattle	17	Morphology	- Flukes that had no sperm in their seminal vesicles or had “very scanty sperm” were found and deemed “aspermic”. - Specimens were grouped into *F. hepatica*, *F. gigantica,* and *Fasciola* sp. based on the body length/width/ratio measurements. - The average length/width and corresponding standard deviations of *F. hepatica*¸ *F. gigantica* and *Fasciola* sp. were 21.16 ± 4.29/10.53 ± 1.80 mm, 39.61 ± 1.09/10.44 ± 1.59 mm and 28.87 ± 5.12/9.32 ± 1.72 mm, respectively.
[105]	To differentiate between the three fasciolid worms encountered in sheep and cattle in Sohag, Egypt, through a simple and rapid PCR-restriction fragment length polymorphism (RFLP) assay, using the common restriction enzymes AvaII based on a 618-bp-long sequence of the 28S rRNA gene.	Egypt	Sheep, cattle	-	Morphology and molecular	- Intermediate forms possessed morphometric characters from *F. hepatica* (length and pattern of uterine coils) however the species was genetically more related to *F. gigantica*.
[106]	To determine the occurrence rate of *Fasciola* spp. in sheep as measured by post-mortem examination of slaughtered animals at abattoirs.	Egypt	Buffalo	2	Molecular	- Two introgressed *Fasciola* forms had ITS-1 sequences identical to *F. hepatica* and mitochondrial NDI sequences identical to *F. gigantica*.
[107]	To determine the prevalence of fascioliasis in cattle, and to describe the histopathological changes in the liver and lungs.	Egypt	Cattle	35	Morphology	- *Fasciola* hepatica possessed an oral sucker equal in size to the ventral sucker, at the conical anterior end and rudimentary inner intestinal branches. *F. gigantica* had an oral sucker that was larger than the ventral sucker at the anterior end and T- and Y-shaped intestinal caeca branches. The intermediate form had “few” of these morphological features from both *F. hepatica* and *F. gigantica*.
[108]	To use sequence analysis of the ITS-2 region of rDNA and highly repetitive DNA sequences to determine the identity and heterogeneity among *Fasciola* isolated from buffalo, cow, and sheep hosts.	Egypt	Sheep	1	Molecular	- Intermediate *Fasciola* isolate had sequence variation in several sites from both *F. hepatica* and *F. gigantica*.
[109]	Analyzed the morphometric characteristics of fasciolid adults infecting the main livestock species present in the Nile Delta human endemic area.	Egypt	Cattle, buffalo	126	Morphology	- Body roundness (BR), Body length/Body width (BL/BW) and the distance between the ventral sucker and the posterior end of the body (VS-P) measurements of intermediate *Fasciola* overlapped between *F. hepatica* and *F. gigantica* measurements.

## Data Availability

Not applicable.

## Supplementary Materials

The following supporting information can be downloaded at https://www.mdpi.com/article/10.3390/pathogens11111303/s1. Table S1: Summary of African studies reporting on the presence of *Fasciola* species recovered from various definitive hosts. Table S2: The distribution and occurrence of *Fasciola* species in Africa based on studies conducted from 1980–2022. Table S3: Summary of African studies reporting on the occurrence of *Fasciola* species in their snail intermediate hosts. Table S4: Summary of studies reporting on the occurrence of intermediate hosts of *Fasciola* spp. in Africa. References [140,141,142,143,144,145,146,147,148,149,150,151,152,153,154,155,156,157,158,159,160,161,162,163,164,165,166,167,168,169,170,171,172,173,174,175,176,177,178,179,180,181,182,183,184,185,186,187,188,189,190,191,192,193,194,195,196,197,198,199,200,201,202,203,204,205,206,207,208,209,210,211,212,213,214,215,216,217,218,219,220,221,222,223,224,225,226,227,228,229,230,231,232,233,234,235,236,237,238,239,240,241,242,243,244,245,246,247,248,249,250,251,252,253,254,255,256,257,258,259,260,261,262,263,264,265,266,267,268,269,270,271,272,273,274,275,276,277,278,279,280,281,282,283,284,285,286,287,288,289,290,291,292,293,294,295,296,297,298,299,300,301,302,303,304,305,306,307,308,309,310,311,312,313,314,315,316,317] are cited in the supplementary materials.

## References

[1] Omar M.A., Elmajdoub L.O., Ali A.O., Ibrahim D.A., Sorour S.S., Al-Wabel M.A., Ahmed A.I., Suresh M., Metwally A.M. (2021). Genetic characterization and phylogenetic analysis of *Fasciola* species based on ITS2 gene sequence, with first molecular evidence of intermediate *Fasciola* from water buffaloes in Aswan, Egypt. Ann. Parasitol..

[2] Giovanoli Evack J., Schmidt R.S., Boltryk S.D., Voss T.S., Batil A.A., Ngandolo B.N., Greter H., Utzinger J., Zinsstag J., Balmer O. (2020). Molecular confirmation of a *Fasciola gigantica*× *Fasciola hepatica* hybrid in a Chadian bovine. J. Parasitol. Res..

[3] Sabourin E., Alda P., Vazquez A., Hurtrez-Bousses S., Vittecoq M. (2018). Impact of Human Activities on Fasciolosis Transmission. Trends Parasitol..

[4] Admassu B., Shite A., Kinfe G. (2015). A review on bovine fasciolosis. Eur. J. Biol. Sci..

[5] Abebe R., Abunna F., Berhane M., Mekuria S., Megersa B., Regassa A. (2010). Fasciolosis: Prevalence, financial losses due to liver condemnation and evaluation of a simple sedimentation diagnostic technique in cattle slaughtered at Hawassa Municipal abattoir southern Ethiopia. Ethiop. Vet. J..

[6] Robinson M.W., Dalton J.P. (2009). Zoonotic helminth infections with particular emphasis on fasciolosis and other trematodiases. Philos. Trans. R. Soc. Lond. B Biol. Sci..

[7] Prasad P., Tandon V., Biswal D.K., Goswami L.M., Chatterjee A. (2008). Molecular identification of the Indian liver fluke, *Fasciola* (Trematoda: Fasciolidae) based on the ribosomal internal transcribed spacer regions. Parasitol. Res..

[8] Rehman Z.U., Martin K., Zahid O., Ali Q., Rashid I., Hafeez M.A., Ahmad N., Ashraf K., Betson M., Sargison N.D. (2021). High-throughput sequencing of *Fasciola* spp. shows co-infection and intermediate forms in Balochistan, but only *Fasciola gigantica* in the Punjab province of Pakistan. Infect. Genet. Evol..

[9] Mas-Coma S., Bargues M.D., Valero M.A. (2005). Fascioliasis and other plant-borne trematode zoonoses. Int. J. Parasitol..

[10] Marcilla A.M., Bargues D., Mas-Coma S. (2002). A PCR-RFLP assay for the distinction between *Fasciola hepatica* and *Fasciola gigantica*. Mol. Cell. Probes.

[11] Mas-Coma S., Valero M.A., Bargues M.D. (2009). *Fasciola*, lymnaeids and human fascioliasis, with a global overview on disease transmission, epidemiology, evolutionary genetics, molecular epidemiology and control. Adv. Parasitol..

[12] Mas-Coma S., Bargues M.D. (1997). Human liver flukes: A review. Res. Rev. Parasitol..

[13] Itagaki T., Hayashi K., Ohari Y. (2022). The causative agents of fascioliasis in animals and humans: Parthenogenetic *Fasciola* in Asia and other regions. Infect. Genet. Evol..

[14] Alemu A. (2019). Prevalence of dairy cattle fasciolosis in and around Wolayta Sodo, Southern Ethiopia. J. Dairy Res. Technol..

[15] Caron Y., Rondelaud D., Losson B. (2008). The detection and quantification of a digenean infection in the snail host with special emphasis on *Fasciola* sp.. Parasitol. Res..

[16] Mahulu A., Clewing C., Stelbrink B., Chibwana F.D., Tumwebaze I., Stothard J.R., Albrecht C. (2019). Cryptic intermediate snail host of the liver fluke *Fasciola hepatica* in Africa. Parasites Vectors.

[17] Dinnik J.A., Dinnik N.N. (1957). A mud snail, *Lymnaea mweruensis* Connolly as an intermediate host of both liver flukes *Fasciola hepatica* and *Fasciola gigantica* Cobbold. Rep. E Afr. Vet. Res. Org..

[18] Goll P.H., Scott J.M. (1978). The interrelationship of *Lymnaea truncatula* and ovine fascioliasis in the Ethiopian central highlands. Br. Vet. J..

[19] Prinsloo J.F., Van Eeden J.A. (1976). Population dynamics of freshwater snails in Lesotho with particular reference to *Lymnaea truncatula*: The intermediate host of *Fasciola hepatica*. Wet. Byd. Potchefstroomse Univ. B Nat..

[20] Lotfy W.M., Brant S.V., DeJong R.J., Le T.H., Demiaszkiewicz A., Rajapakse R.P., Perera V.B., Laursen J.R., Loker E.S. (2008). Evolutionary origins, diversification, and biogeography of liver flukes (Digenea, Fasciolidae). Am. J. Trop. Med. Hyg..

[21] Brown D. (1994). Freshwater Snails of Africa and Their Medical Importance.

[22] Malatji M.P., Pfukenyi D.M., Mukaratirwa S. (2019). *Fasciola* species and their vertebrate and snail intermediate hosts in East and Southern Africa: A review. J. Helminthol..

[23] Malatji M., Mukaratirwa S. (2019). Molecular detection of natural infection of *Lymnaea* (*Pseudosuccinea*) *columella* (Gastropoda: Lymnaeidae) with *Fasciola gigantica* (Digenea: Fasciolidae) from two provinces of South Africa. J. Helminthol..

[24] De Kock K.N., Joubert P.H., Pretorius S.J. (1989). Geographical distribution and habitat preferences of the invader freshwater snail species *Lymnaea columella* (Mollusca: Gastropoda) in South Africa. Onderstepoort J. Vet. Res..

[25] Alba A., Vázquez A.A., Sánchez J., Duval D., Hernández H.M., Sabourin E., Vittecoq M., Hurtrez-Boussés S., Gourbal B. (2018). *Fasciola hepatica*–*Pseudosuccinea columella* interaction: Effect of increasing parasite doses, successive exposures and geographical origin on the infection outcome of susceptible and naturally resistant snails from Cuba. Parasites Vectors.

[26] Grabner D.S., Mohamed F.A., Nachev M., Méabed E.M., Sabry A.H., Sures B. (2014). Invasion biology meets parasitology: A case study of parasite spill-back with Egyptian *Fasciola gigantica* in the invasive snail *Pseudosuccinea columella*. PLoS ONE.

[27] Dar Y., Vignoles P., Rondelaud D., Dreyfuss G. (2015). Role of the lymnaeid snail *Pseudosuccinea columella* in the transmission of the liver fluke *Fasciola hepatica* in Egypt. J. Helminthol..

[28] Carolus H., Muzarabani K.C., Hammoud C., Schols R., Volckaert F.A.M., Barson M., Huyse T. (2019). A cascade of biological invasions and parasite spillback in man-made Lake Kariba. Sci. Total. Environ..

[29] Amer S., Dar Y., Ichikawa M., Fukuda Y., Tada C., Itagaki T., Nakai Y. (2011). Identification of *Fasciola* species isolated from Egypt based on sequence analysis of genomic (ITS1 and ITS2) and mitochondrial (NDI and COI) gene markers. Parasitol. Int..

[30] Haridwal S., Malatji M.P., Mukaratirwa S. (2021). Morphological and molecular characterization of *Fasciola hepatica* and *Fasciola gigantica* phenotypes from co-endemic localities in Mpumalanga and KwaZulu-Natal provinces of South Africa. Food Waterborne Parasitol..

[31] Calvani N.E.D., Ichikawa-Seki M., Bush R.D., Khounsy S., Slapeta J. (2020). Which species is in the faeces at a time of global livestock movements: Single nucleotide polymorphism genotyping assays for the differentiation of *Fasciola* spp.. Int. J. Parasitol..

[32] Nguyen T.B.N., Van De N., Nguyen T.K.L., Quang H.H., Doan H.T.T., Agatsuma T., Le T.H. (2018). Distribution Status of Hybrid Types in Large Liver Flukes, *Fasciola* Species (Digenea: Fasciolidae), from Ruminants and Humans in Vietnam. Korean J. Parasitol..

[33] Watanabe S., Iwata S. (1954). Various *Fasciola* genus in Japan. J. Jpn. Vet. Med. Assoc..

[34] Huang W.Y., He B., Wang C.R., Zhu X.Q. (2004). Characterisation of *Fasciola* species from Mainland China by ITS-2 ribosomal DNA sequence. Vet. Parasitol..

[35] Arksey H., O’Malley L. (2005). Scoping studies: Towards a Methodological Framework. Int. J. Soc. Res. Methodol..

[36] Righi S., Benakhla A., Mekroud A., Ouchene N., Sedraoui S. (2016). Prevalence of *Fasciola hepatica* in *Galba truncatula* detected by multiplex PCR in the province of El Tarf (Algeria). Trop. Biomed..

[37] Mekroud A., Benakhla A., Vignoles P., Rondelaud D., Dreyfuss G. (2004). Preliminary studies on the prevalences of natural fasciolosis in cattle, sheep, and the host snail (*Galba truncatula*) in north-eastern Algeria. Parasitol. Res..

[38] Arafa W., Hassan A., Snousi S., El-Dakhly K.M., Holman P., Craig T., Aboelhadid S. (2017). *Fasciola hepatica* infections in cattle and the freshwater snail *Galba truncatula* from Dakhla Oasis, Egypt. J. Helminthol..

[39] Dar Y., Amer S., Eddine R.Z., Dreyfuss G. (2016). Characterisation of *Pseudosuccinea columella* and *Radix natalensis* (Gastropoda: Lymnaeidae) in Egypt using shell and molecular data. Molluscan Res..

[40] Arafa W.M. (2015). Detection of *Fasciola hepatica* infection in cattle and *Lymnaea truncatula* snails in Dakhla Oasis, Egypt. Egypt. Vet. Med. Soc. Parasitol. J. EVMSPJ.

[41] Abd El-Wakeil K.F., Obuid-Allah A.H., Mohamed A.H., Abd El-Aziz F.E.-Z.A. (2013). Community structure of molluscans in River Nile and its branches in Assiut governorate, Egypt. Egypt. J. Aquat. Res..

[42] El-Shazly A.M., Nabih N., Salem D.A., Mohamed M.Z. (2012). Snail populations in Dakahlia Governorate, Egypt, with special reference to lymnaeids. Egypt. J. Biol..

[43] Dar Y., Djuikwo Teukeng F.F., Vignoles P., Dreyfuss G., Rondelaud D. (2010). *Radix natalensis*, a potential intermediate host of *Fasciola hepatica* in Egypt. Parasite.

[44] Ibrahim M., Shalaby I., Salim I.I. (2006). Freshwater Snails and Larval Trematode Communities In El-Salaam Irrigation Canal, North Sinai, Egypt. Egypt. J. Zool..

[45] El-Shazly A.M., Helmy M.M., Haridy F.M., El-Sharkawy E.M., Morsy T.A. (2002). *Fasciola* immature stages sought in *Lymnaea* species and *Biomphalaria* species in the water bodies of Dakahlia Governorate. J. Egypt. Soc. Parasitol..

[46] El-Kady G.A., Shoukry A., Reda L.A., El-Badri Y.S. (2000). Survey and population dynamics of freshwater snails in newly settled areas of the Sinai Peninsula. Egypt. J. Biol..

[47] Ahmed A.H., Ramzy R.M. (1999). Infection of two lymnaeid snails with *Fasciola gigantica* in Giza, a field study. J. Egypt. Soc. Parasitol..

[48] Farag H., El Sayad M. (1995). *Biomphalaria alexandrina* naturally infected with *Fasciola gigantica* in Egypt. Trans. R. Soc. Trop. Med. Hyg..

[49] Sattmann H., Kinzelbah R. (1988). Notes on inland water molluscs from Egypt (Mollusca: Gastropoda, Bivalvia). Zool. Middle. East..

[50] Khallaayoune K., Stromberg B.E., Dakkak A., Malone J.B. (1991). Seasonal dynamics of *Fasciola hepatica* burdens in grazing Timahdit sheep in Morocco. Int. J. Parasitol..

[51] Hamed N., Ayadi A., Hammami H. (2014). Epidemiological studies on fasciolosis in northern Tunisia. Rev. Méd. Vét..

[52] Hamed N., Hammami H., Khaled S., Rondelaud D., Ayadi A. (2009). Natural infection of *Fasciola hepatica* (Trematoda: Fasciolidae) in *Bulinus truncatus* (Gastropoda: Planorbidae) in northern Tunisia. J. Helminthol..

[53] Hammami H., Hamed N., Ayadi A. (2007). Epidemiological studies on *Fasciola hepatica* in Gafsa Oases (southwest of Tunisia). Parasite.

[54] Tchakonté S., Ajeagah G.A., Diomandé D., Camara A.I., Ngassam P. (2014). Diversity, dynamic and ecology of freshwater snails related to environmental factors in urban and suburban streams in Douala–Cameroon (Central Africa). Aquat. Ecol..

[55] Malatji M.P., Lamb J., Mukaratirwa S. (2019). Molecular characterization of liver fluke intermediate host lymnaeids (Gastropoda: Pulmonata) snails from selected regions of Okavango Delta of Botswana, KwaZulu-Natal and Mpumalanga provinces of South Africa. Vet. Parasitol. Reg. Stud. Rep..

[56] Appleton C.C., Curtis B.A., Alonso L.E., Kipping J., Alonso L.E., Nordin L.A. (2003). Freshwater invertebrates of the Okavango Delta, Botswana. A rapid Biological Assessment of the Aquatic Ecosystems of the Okavango Delta, Botswana: High Water Survey.

[57] De Kock K.N., Wolmaran C.T., Bornman M. (2003). Distribution and habitats of the snail *Lymnaea truncatula*, intermediate host of the liver fluke *Fasciola hepatica*, in South Africa. J. S. Afr. Vet. Assoc..

[58] Curtis B.A. (1991). Freshwater macro-invertebrates of Namibia. Madoqua.

[59] Akamagwuna F.C., Odume O.N., Richoux N.B. (2022). Exploring the community structure of Afrotropical macroinvertebrate traits and ecological preferences along an agricultural pollution gradient in the Kat River, Eastern Cape, South Africa. Ecol. Indic..

[60] Erasmus J.H., Lorenz A.W., Zimmermann S., Wepener V., Sures B., Smit N.J., Malherbe W. (2021). A diversity and functional approach to evaluate the macroinvertebrate responses to multiple stressors in a small subtropical austral river. Ecol. Indic..

[61] Moema E.B., King P.H., Rakgole J.N. (2019). Phylogenetic studies of larval digenean trematodes from freshwater snails and fish species in the proximity of Tshwane metropolitan, South Africa. Onderstepoort J. Vet. Res..

[62] Lounnas M., Correa A.C., Vázquez A.A., Dia A., Escobar J.S., Nicot A., Arenas J., Ayaqui R., Dubois M.P., Gimenez T. (2017). Self-fertilization, long-distance flash invasion and biogeography shape the population structure of *Pseudosuccinea columella* at the worldwide scale. Mol. Ecol..

[63] Kemp M., de Kock K.N., Zaayman J.L., Wolmarans C.T. (2016). A comparison of mollusc diversity between the relatively pristine Marico River and the impacted Crocodile River, two major tributaries of the Limpopo River, South Africa. Water SA.

[64] Appleton C.C., Miranda N.A.F. (2015). Two Asian freshwater snails newly introduced into South Africa and an analysis of alien species reported to date. Afr. Invertebr..

[65] Wolmarans C., Wepener V., Pretorius U., Erasmus J., de Kock K. (2015). A comparison of the Mollusca diversity in the Mooi River (North-West Province) as found during surveys conducted in 1963 and again 50 years later. S. Afr. Tydskr. Natuurwet. Tegnol..

[66] Perissinotto R., Miranda N.A.F., Raw J.L., Peer N. (2014). Biodiversity census of lake St Lucia, iSimangaliso wetland park (South Africa): Gastropod molluscs. Zookeys.

[67] De Kock K., Wolmarans C., Kemp M., Roets W. (2013). Short-term threats for the sustained survival of freshwater Mollusca in the Olifants River and selected tributaries. S. Afr. Tydskr. Natuurwet. Tegnol..

[68] Moema E.B.E., King P.H., Baker C. (2012). Descriptions of strigea cercariae from the Gauteng and Northwest Proinces, South Africa. Onderstepoort J. Vet. Res..

[69] Miranda N.A., Perissinotto R., Appleton C.C. (2011). Population structure of an invasive parthenogenetic gastropod in coastal lakes and estuaries of northern KwaZulu-Natal, South Africa. PLoS ONE.

[70] De Kock K.N., Wolmarans C.T. (2008). Invasive alien freshwater snail species in the Kruger National Park, South Africa. Koedoe.

[71] Moema E., King P.H., Baker C. (2008). Cercariae developing in *Lymnaea natalensis* Krauss, 1848 collected in the vicinity of Pretoria, Gauteng Province, South Africa. Onderstepoort J. Vet. Res..

[72] Wolmarans C., De Kock K.N. (2006). The current status of freshwater molluscs in the Kruger National Park. Koedoe.

[73] De Kock K.N., Wolmarans C.T., du Preez L.H. (2002). Freshwater mollusc diversity in the Kruger National Park: A comparison between a period of prolonged drought and a period of exceptionally high rainfall. Koedoe.

[74] De Kock K.N., Wolmarans C.T., Strauss H.D., Killian M. (2001). Distribution, and habitats of *Lymnaea natalensis*, snail intermediate host of the liver fluke *Fasciola gigantica*, in South Africa. S. Afr. J. Sci. Tech..

[75] De Kock K.N., Wolmarans C.T. (1998). A re-evaluation of the occurrence of freshwater molluscs in the Kruger National Park. Koedoe.

[76] Wilken G.B., Appleton C.C. (1991). Avoidance responses of some indigenous and exotic freshwater pulmonate snails to leech predation in South Africa. S. Afr. J. Zool..

[77] Phiri A., Mudenda N., Luwe M., Phiri I. (2017). Use of bait containing triclabendazole against *Fasciola gigantica* in a herd of captive wild impala (*Aepyceros melampus*). J. Helminthol..

[78] Phiri A., Phiri I.K., Chota A., Monrad J. (2007). Trematode infections in freshwater snails and cattle from the Kafue wetlands of Zambia during a period of highest cattle–water contact. J. Helminthol..

[79] Schols R., Carolus H., Hammoud C., Muzarabani K.C., Barson M., Huyse T. (2021). Invasive snails, parasite spillback, and potential parasite spillover drive parasitic diseases of *Hippopotamus amphibius* in artificial lakes of Zimbabwe. BMC Biol..

[80] Pfukenyi D., Mukaratirwa S., Willingham A.L., Monrad J. (2006). Epidemiological studies of *Fasciola gigantica* infections in cattle in the highveld and lowveld communal grazing areas of Zimbabwe. Onderstepoort J. Vet. Res..

[81] Chingwena G., Mukaratirwa S., Kristensen T.K., Chimbari M. (2002). Susceptibility of Freshwater Snails to the Amphistome *Calicophoron microbothrium* and the Influence of the Species on Susceptibility of *Bulinus tropicus* to *Schistosoma haematobium* and *Schistosoma mattheei* Infections. J. Parasitol..

[82] Ibikounlé M., Ogouyèmi-Hounto A., Sissinto Savi de Tové Y., Dansou A., Courtin D., Kindé-Gazard D., Mouahid G., Moné H., Massougbodji A. (2014). Epidemiology of urinary schistosomiasis among school children in Péhunco area, Northern Benin. Malacological survey. Bull. Soc. Pathol. Exot..

[83] Ibikounlé M., Mouahid G., Sakiti N.G., Massougbodji A., Moné H. (2009). Freshwater snail diversity in Benin (West Africa) with a focus on human schistosomiasis. Acta Trop..

[84] Koné K., Bony K.Y., Konan K.F., Edia O.E., Gnagne T., Gourène G. (2013). Freshwater snail dynamics focused on potential risk of using urine as fertilizer in Katiola, an endemic area of Schistosomiasis (Ivory Coast; West Africa). J. Entomol. Zool. Stud..

[85] Rabone M., Wiethase J.H., Allan F., Gouvras A.N., Pennance T., Hamidou A.A., Webster B.L., Labbo R., Emery A.M., Garba A.D. (2019). Freshwater snails of biomedical importance in the Niger River Valley: Evidence of temporal and spatial patterns in abundance, distribution and infection with *Schistosoma* spp.. Parasites Vectors.

[86] Oso O.G., Odaibo A.B. (2021). Land use/land cover change, physico-chemical parameters and freshwater snails in Yewa North, Southwestern Nigeria. PLoS ONE.

[87] Gadzama I., Ezealor A.U., Aken’Ova T.O., Balarabe M.L. (2015). Aspects of the Geomorphology and Limnology of some mollusc-inhabited freshwater bodies in northern Nigeria. IOSR J. Environ. Sci. Toxicol. Food Technol..

[88] Hamlili F.Z., Thiam F., Laroche M., Diarra A.Z., Doucoure’ S., Gaye P.M., Fall C.B., Faye B., Sokhna C., Sow D. (2021). MALDITOF mass spectrometry for the identification of freshwater snails from Senegal, including intermediate hosts of schistosomes. PLoS Negl. Trop. Dis..

[89] Olkeba B.K., Boets P., Mereta S.T., Yeshigeta M., Geremew Muleta Akessa G.M., Argaw Ambelu A., Goethals P.L.M. (2020). Environmental and biotic factors affecting freshwater snail intermediate hosts in the Ethiopian Rift Valley region. Parasites Vectors.

[90] Yigezu G., Mandefro B., Mengesha Y., Yewhalaw D., Beyene A., Ahmednur M., Abdie Y., Kloos H., Mereta S.T. (2018). Habitat suitability modelling for predicting potential habitats of freshwater snail intermediate hosts in Omo-Gibe river basin, Southwest Ethiopia. Ecol. Inform..

[91] Mandefro B., Mereta S.T., Tariku Y., Ambelu A. (2017). Molluscicidal effect of *Achyranthes aspera* L. (Amaranthaceae) aqueous extract on adult snails of *Biomphalaria pfeifferi* and *Lymnaea natalensis*. Infect. Dis. Poverty.

[92] Tadesse E., Tilahun G., Girmay M. (2006). Some biological properties of *Lymnaea truncatula* and production of *Fasciola hepatica* metacercariae. Eth. J. Sci..

[93] Stothard J.R., Brémond P., Andriamaro L., Loxton N.J., Sellin B., Sellin E., Rollinson D. (2000). Molecular characterization of the freshwater snail *Lymnaea natalensis* (Gastropoda: Lymnaeidae) on Madagascar with an observation of an unusual polymorphism in ribosomal small subunit genes. J. Zool. Lond..

[94] Da Costa C., Dreyfuss G., Rakotondravao O., Rondelaud D. (1994). Several observations concerning cercarial sheddings of *Fasciola gigantica* from *Lymnaea natalensis*. Parasite.

[95] Outa J.O., Sattmann H., Köhsler M., Walochnik J., Jirsa F. (2020). Diversity of digenean trematode larvae in snails from Lake Victoria, Kenya: First reports and bioindicative aspects. Acta Trop..

[96] Owiny M.O., Obonyo M.O., Gatongi P.M., Fèvre E.M. (2019). Prevalence and spatial distribution of Trematode cercariae in Vector Snails within different Agro-Ecological Zones in Western Kenya, 2016. Pan. Afr. Med. J..

[97] Dida G.O., Gelder F.B., Anyona D.N., Matano A.S., Abuom P.O., Adoka S.O., Ouma C., Kanangire C.K., Owuor P.O., Ofulla A.V. (2014). Distribution and abundance of schistosomiasis and fascioliasis host snails along the Mara River in Kenya and Tanzania. Infect. Ecol. Epidemiol..

[98] Wamae L., Ongare J., Ihiga M., Mahaga M. (1990). Epidemiology of fasciolosis on a ranch in the central Rift Valley, Kenya. Trop. Anim. Health. Prod..

[99] Wamae L., Cheruiyot H. (1990). Incidence of *Fasciola gigantica* intramolluscan stages in *Lymnaea natalensis*, the intermediate host, over a one-year period in Kenya. Bull. Anim. Health Prod. Afr..

[100] Howell A., Mugisha L., Davies J., LaCourse E.J., Claridge J., Williams D.J., Kelly-Hope L., Betson M., Kabatereine N.B., Stothard J.R. (2012). Bovine fasciolosis at increasing altitudes: Parasitological and malacological sampling on the slopes of Mount Elgon, Uganda. Parasites Vectors.

[101] Stensgaard A.S., Jørgensen A., Kabatereine N.B., Rahbek C., Kristensen T.K. (2006). Modeling freshwater snail habitat suitability and areas of potential snail-borne disease transmission in Uganda. Geospat. Health.

[102] Nzalawahe J., Kassuku A.A., Stothard J.R., Coles G.C., Eisler M.C. (2014). Trematode infections in cattle in Arumeru District, Tanzania are associated with irrigation. Parasites Vectors.

[103] Walker S., Makundi A., Namuba F., Kassuku A., Keyyu J., Hoey E., Prödohl P., Stothard J.R., Trudgett A. (2008). The distribution of *Fasciola hepatica* and *Fasciola gigantica* within southern Tanzania–constraints associated with the intermediate host. Parasitology.

[104] Utzinger J., Tanner M. (2000). Microhabitat preferences of *Biomphalaria pfeifferi* and *Lymnaea natalensis* in a natural and a man-made habitat in southeastern Tanzania. Mem. Inst. Oswaldo Cruz.

[105] Khalifa R., El-Hady H.A., Omran E.K., Ahmed N.S. (2013). Genetically confirmed *Fasciola hepatogigantica* n. sp.. J. Egypt. Soc. Parasitol..

[106] Amer S., ElKhatam A., Zidan S., Feng Y., Xiao L. (2016). Identity of *Fasciola* spp. in sheep in Egypt. Parasites Vectors.

[107] Sotohy A.S., Abdallah A.H., Wafaa G.M., Abeer A.K. (2019). Prevalence and histopathological changes of bovine fascioliasis, with unusual migration to lung in new-valley governorate. Assiut. Vet. Med. J..

[108] Taha H.A. (2014). Molecular Characterization of *Fasciola* spp. in Egypt on the Basis of certain rDNA fragments and Highly Repetitive DNA Sequences. Egypt. Acad. J. Biol. Sci..

[109] Periago M.V., Valero M.A., El Sayed M., Ashrafi K., El Wakeel A., Mohamed M.Y., Desquesnes M., Curtale F., Mas-Coma S. (2008). First phenotypic description of *Fasciola hepatica*/*Fasciola gigantica* intermediate forms from the human endemic area of the Nile Delta. Egypt. Infect. Genet. Evol..

[110] Dermauw V., Muchai J., Al Kappany Y., Fajardo Castaneda A.L., Dorny P. (2021). Human fascioliasis in Africa: A systematic review. PLoS ONE.

[111] Chougar L., Mas-Coma S., Artigas P., Harhoura K., Aissi M., Agramunt V.H., Bargues M.D. (2020). Genetically ‘pure’ *Fasciola gigantica* discovered in Algeria: DNA multimarker characterization, trans-Saharan introduction from a Sahel origin and spreading risk into north-western Maghreb countries. Transbound. Emerg. Dis..

[112] Mehmood K., Zhang H., Sabir A.J., Abbas R.Z., Ijaz M., Durrani A.Z., Saleem M.H., Ur Rehman M., Iqbal M.K., Wang Y. (2017). A review on epidemiology, global prevalence and economical losses of fasciolosis in ruminants. Microb. Pathog..

[113] Khanjari A., Bahonar A., Fallah S., Bagheri M., Alizadeh A., Fallah M., Khanjari Z. (2014). Prevalence of fasciolosis and dicrocoeliosis in slaughtered sheep and goats in Amol Abattoir, Mazandaran, northern Iran. Asian Pac. J. Trop. Dis..

[114] Garcia-Campos A., Correia C.N., Naranjo-Lucena A., Garza-Cuartero L., Farries G., Browne J.A., MacHugh D.E., Mulcahy G. (2019). *Fasciola hepatica* Infection in Cattle: Analyzing Responses of Peripheral Blood Mononuclear Cells (PBMC) Using a Transcriptomics Approach. Front. Immunol..

[115] Cwiklinski K., O’Neill S.M., Donnelly S., Dalton J.P. (2016). A prospective view of animal and human Fasciolosis. Parasite Immunol..

[116] Munyeme M., Muma J.B., Skjerve E., Nambota A.M., Phiri I.G.K., Samui K.L., Dorny P., Tryland M. (2008). Risk factors associated with bovine tuberculosis in traditional cattle of the livestock/wildlife interface areas in the Kafue basin of Zambia. Prev. Vet. Med..

[117] Boomker J. (2007). Helminth Infections: Wildlife.

[118] Muma J., Samui K.L., Oloya J., Munyeme M., Skjerve E. (2007). Risk factors for brucellosis in indigenous cattle reared in livestock–wildlife interface areas of Zambia. Prev. Vet. Med..

[119] Nyagura I., Malatji M.P., Mukaratirwa S. (2022). Occurrence of *Fasciola* (Digenea: Fasciolidae) Species in Livestock, Wildlife and Humans, and the Geographical Distribution of Their Intermediate Hosts in South Africa—A Scoping Review. Front. Vet. Sci..

[120] Black J., Ntusi N., Stead P., Mayosi B., Mendelson M. (2013). Human fascioliasis in South Africa. S. Afr. Med. J..

[121] Soliman M.F. (2008). Epidemiological review of human and animal fascioliasis in Egypt. J. Infect. Dev. Ctries..

[122] Abe E.M., Guo Y.H., Shen H., Mutsaka-Makuvaza M.J., Habib M.R., Xue J.B., Midzi N., Xu J., Li S.Z., Zhou X.N. (2018). Phylogeography of Bulinus truncatus (Audouin, 1827) (Gastropoda: Planorbidae) in Selected African Countries. Trop. Med. Infect. Dis..

[123] Ichikawa-Seki M., Peng M., Hayashi K., Shoriki T., Mohanta U.K., Shibahara T., Itagaki T. (2017). Nuclear and mitochondrial DNA analysis reveals that hybridization between *Fasciola hepatica* and *Fasciola gigantica* occurred in China. Parasitology.

[124] Peng M., Ichinomiya M., Ohtori M., Ichikawa M., Shibahara T., Itagaki T. (2009). Molecular characterization of *Fasciola hepatica*, *Fasciola gigantica*, and aspermic *Fasciola* sp. in China based on nuclear and mitochondrial DNA. Parasitol. Res..

[125] Nguyen S., Amer S., Ichikawa M., Itagaki T., Fukuda Y., Nakai Y. (2012). Molecular identification of *Fasciola* spp. (Digenea: Platyhelminthes) in cattle from Vietnam. Parasite.

[126] Itagaki T., Sakaguchi K., Terasaki K., Sasaki O., Yoshihara S., Van Dung T. (2009). Occurrence of spermic diploid and aspermic triploid forms of *Fasciola* in Vietnam and their molecular characterization based on nuclear and mitochondrial DNA. Parasitol. Int..

[127] Nguyen T.G., Van De N., Vercruysse J., Dorny P., Le T.H. (2009). Genotypic characterization and species identification of *Fasciola* spp. with implications regarding the isolates infecting goats in Vietnam. Exp. Parasitol..

[128] Le T.H., Nguyen V., Agatsuma T., Nguyen T.G.T., Nguyen Q.D., McManus D.P., Blair D. (2008). Human fascioliasis and the presence of hybrid/introgressed forms of *Fasciola hepatica* and *Fasciola gigantica* in Vietnam. Parasitol. Int..

[129] Ohari Y., Sato H., Nonaka N., Mohanta U., Hayashi K., Itagaki T. (2016). Genetic characterization of *Fasciola* flukes detected from wild sika deer in Hokkaido, Yamaguchi and Miyazaki prefectures, Japan. Jpn. J. Vet. Parasitol..

[130] Itagaki T., Kikawa M., Sakaguchi K., Shimo J., Terasaki K., Shibahara T., Fukuda K. (2005). Genetic characterization of parthenogenic *Fasciola* sp. in Japan on the basis of the sequences of ribosomal and mitochondrial DNA. Parasitology.

[131] Itagaki T., Kikawa M., Terasaki K., Shibahara T., Fukuda K. (2005). Molecular characterization of parthenogenic *Fasciola* sp. in Korea on the basis of DNA sequence of ribosomal ITS1 and mitochondrial NDI gene. J. Vet. Med. Sci..

[132] Agatsuma T., Arakawa Y., Iwagami M., Honzako Y., Cahyaningsih U., Kang S.Y., Hong S.J. (2000). Molecular evidence of natural hybridization between *Fasciola hepatica* and *F. gigantica*. Parasitol. Int..

[133] Mohanta U.K., Ichikawa-Seki M., Shoriki T., Katakura K., Itagaki T. (2014). Characteristics and molecular phylogeny of *Fasciola* flukes from Bangladesh, determined based on spermatogenesis and nuclear and mitochondrial DNA analyses. Parasitol. Res..

[134] Shoriki T., Ichikawa-Seki M., Devkota B., Rana H.B., Devkota S.P., Humagain S.K., Itagaki T. (2014). Molecular phylogenetic identification of *Fasciola* flukes in Nepal. Parasitol. Int..

[135] Ichikawa M., Bawn S., Maw N.N., Htun L.L., Thein M., Gyi A., Sunn K., Katakura K., Itagaki T. (2011). Characterization of *Fasciola* spp. in Myanmar on the basis of spermatogenesis status and nuclear and mitochondrial DNA markers. Parasitol. Int..

[136] Okamoto E., Tashiro M., Ortiz P., Mohanta U.K., Hobán C., Murga-Moreno C.A., Angulo-Tisoc J.M., Ichikawa-Seki M. (2022). Development of novel DNA marker for species discrimination of *Fasciola* flukes based on the fatty acid binding protein type I gene. Parasites Vectors.

[137] Hashimoto K., Watanobe T., Liu C.X., Init I., Blair D., Ohnishi S., Agatsuma T. (1997). Mitochondrial DNA and nuclear DNA indicate that the Japanese *Fasciola* species is *F. gigantica*. Parasitol. Res..

[138] Itagaki T., Tsutsumi K.I., Sakamoto T., Tsutsumi Y., Itagaki H. (1995). Characterization of genetic divergence among species within the genus *Fasciola* by PCR-SSCP. Jpn J. Parasitol..

[139] Itagaki T., Tsutsumi K.I., Ito K., Tsutsumi Y. (1998). Taxonomic status of the Japanese triploid forms of *Fasciola*: Comparison of mitochondrial ND1 and COI sequences with *F. hepatica* and *F. gigantica*. J. Parasitol..

[140] Laatamna A.E., Tashiro M., Zokbi Z., Chibout Y., Megrane S., Mebarka F., Ichikawa-Seki M. (2021). Molecular characterization and phylogenetic analysis of *Fasciola hepatica* from high-plateau and steppe areas in Algeria. Parasitol. Int..

[141] Meguini M.N., Righi S., Bouchekhchoukh M., Sedraoui S., Benakhla A. (2021). Investigation of flukes (*Fasciola hepatica* and *Paramphistomum* sp.) parasites of cattle in north-eastern Algeria. Ann. Parasitol..

[142] Amor N., Farjallah S., Merella P., Alagaili A.N., Mohammed O.B. (2020). Multilocus approach reveals discordant molecular markers and corridors for gene flow between North African populations of *Fasciola hepatica*. Vet. Parasitol..

[143] Chaouadi M., Harhoura K., Aissi M., Zait H., Zenia S., Tazerouti F. (2019). A post-mortem study of bovine fasciolosis in the Mitidja (north center of Algeria): Prevalence, risk factors, and comparison of diagnostic methods. Trop. Anim. Health Prod..

[144] Chougar L., Amor N., Farjallah S., Harhoura K., Aissi M., Alagaili A.N., Merella P. (2019). New insight into genetic variation and haplotype diversity of *Fasciola hepatica* from Algeria. Parasitol. Res..

[145] Ouchene-Khelifi N., Ouchene N., Dahmani H., Dahmani A., Sadi M., Douifi M. (2018). Fasciolosis due to *Fasciola hepatica* in ruminants in abattoirs and its economic impact in two regions in Algeria. Trop. Biomed..

[146] Taibi A., Aissi M., Harhoura K., Zenia S., Zait H., Hamrioui B. (2019). Evaluation of *Fasciola hepatica* infections in cattle in northeastern Algeria and the effects on both enzyme and hepatic damage, confirmed by scanning electron microscopy. Acta Parasitol..

[147] Farjallah S., Sanna D., Amor N., Ben Mehel B., Piras M.C., Merella P., Casu M., Curini-Galletti M., Said K., Garippa G. (2009). Genetic characterization of *Fasciola hepatica* from Tunisia and Algeria based on mitochondrial and nuclear DNA sequences. Parasitol. Res..

[148] Abdelazeem A.G., Abdelaziz A.R., Khalafalla R.E., Abushahba M.F.N. (2020). Prevalence and Phylogenetic analysis of *Fasciola* species in Upper Egypt Based on Ribosomal ITS-2 gene Sequencing. Egypt. Vet. Med. Soc. Parasitol. J..

[149] Nasreldin N., Zaki R.S. (2020). Biochemical and immunological investigation of fascioliasis in cattle in Egypt. Vet. World..

[150] El-Tahawy A.S., Kwan N., Sugiura K. (2018). *Fasciola hepatica* infection in water buffalo Bubalus bubalis in three provinces of the Nile Delta, Egypt: A cross-sectional study. J. Vet. Med. Sci..

[151] Ichikawa-Seki M., Tokashiki M., Opara M.N., Iroh G., Hayashi K., Kumar U.M., Itagaki T. (2017). Molecular characterization and phylogenetic analysis of *Fasciola gigantica* from Nigeria. Parasitol. Int..

[152] Khalifa R., Hassanin A., Monib M., Yones D.A., EL-Ossily N., Abdel-Rahman A.S. (2016). Molecular and Phylogenic Characterization of *Fasciola hepatica* from Assiut, Egypt based on nuclear ribosomal DNA sequences. J. Med. Sci. Clin. Res..

[153] Ayoub M.B., Wahba A., Ibrahim M. (2015). Molecular characterization of *Fasciola* spp. in sheep and cattle. Anim. Health Res. Rev..

[154] Badawy A., Abouzaid N., Merwad A. (2014). Occurrence of zoonotic fascioliasis in donkeys in Egypt with emphasis on PCR-RFLP of 28S rRNA gene. Rev. Med. Vet..

[155] Dar Y., Amer S., Mercier A., Courtioux B., Dreyfuss G. (2012). Molecular identification of *Fasciola* spp. (digenea: Fasciolidae) in Egypt. Parasite.

[156] El-Dakhly K.H.M., Abo El-Hadid S.H.M., El-Askalany M.A., Yanai T. (2012). An Abattoir-Based Study on Helminths of Slaughtered Sheep in Beni-Suef, Egypt. Beni Suef Univ. J. Basic Appl. Sci..

[157] El-Rahimy H.H., Mahgoub A., El-Gebaly N.S.M., Mousa W., Antably A.S. (2012). Molecular, biochemical, and morphometric characterization of *Fasciola* species potentially causing zoonotic disease in Egypt. Parasitol. Res..

[158] Walker S.M., Prodöhl P.A., Hoey E.M., Fairweather I., Hanna R.E.B., Brennan G., Trudgett A. (2012). Substantial genetic divergence between morphologically indistinguishable populations of *Fasciola* suggests the possibility of cryptic speciation. Int. J. Parasitol..

[159] Dar Y., Amer S., Courtioux B., Dreyfuss G. (2011). Microsatellite analysis of *Fasciola* spp. in Egypt. Parasitol. Res..

[160] Hussein A.N.A., Hassan I.M., Khalifa R.M.A. (2010). Development, and hatching mechanism of *Fasciola* eggs, light and scanning electron microscopic studies. Saudi J. Biol. Sci..

[161] Hussein A.N.A., Khalifa R.M.A. (2010). Phenotypic description and prevalence of *Fasciola* species in Qena Governorate, Egypt with special reference to a new strain of *Fasciola hepatica*. J. King Saud Univ. Sci..

[162] Valero M.A., Perez-Crespo I., Periago M.V., Khoubbane M., Mas-Coma S. (2009). Fluke egg characteristics for the diagnosis of human and animal fascioliasis by *Fasciola hepatica* and *F. gigantica*. Acta Trop..

[163] Lotfy W.M., El-Morshedy H.N., Abou El-Hoda M., El-Tawila M.M., Omar E.A., Farag H.F. (2002). Identification of the Egyptian species of *Fasciola*. Vet. Parasitol..

[164] El-Azazy O., Fayek S. (1990). Seasonal pattern of *Fasciola gigantica* and *Cysticercus tenuicollis* infections in sheep and goats in Egypt. Bull. Anim. Health Prod. Afr..

[165] Pandey V.S. (1983). Observations on *Fasciola hepatica* in donkeys from Morocco. Ann. Trop. Med. Parasitol..

[166] Mousa A.A., Khitma H., Ochi E.B. (2013). Prevalence and Monetary Loss due to Bovine Fasciolosis in Juba Slaughter House South Sudan. Nat. Sci..

[167] McGarry J., Ortiz P., Hodgkinson J.E., Goreish I., Williams D.J. (2007). PCR-based differentiation of *Fasciola* species (Trematoda: Fasciolidae), using primers based on RAPD-derived sequences. Ann. Trop. Med. Parasitol..

[168] Koko W., Abdalla H., Galal M. (2003). Caprine fascioliasis in the Gezira State, Central Sudan. J. Anim. Vet. Adv..

[169] Koko W., Gala M., Abdalla H. (2003). Gastrointestinal parasites of the Gezira goats: Central Sudan. J. Anim. Vet. Adv..

[170] Amor N., Farjallah S., Said K., Slimane B.B. (2011). First report of *Fasciola hepatica* in *Equus caballus* host species from Tunisia based on the ribosomal internal transcribed spacer regions. Turk. J. Vet. Anim. Sci..

[171] Abass C.G., Abdoulmoumini M., Koumai P., Hervé A.S., Marie W., Mbida M. (2020). Hepatic and Rumenal Worms Infestations of Cattle in Vina Division (Adamawa–Cameroon). Int. J. For. Anim. Fish. Res..

[172] Kelly R., Mazeri S., Hartley C., Hamman S., Ngu Ngwa V., Nkongho E., Tanya V., Sander M., Ndip L., Morgan K. (2019). Assessing the performance of a *Fasciola gigantica* serum antibody ELISA to estimate prevalence in cattle in Cameroon. BMC Vet. Res..

[173] Jean-Richard V., Crump L., Abicho A.A., Naré N.B., Greter H., Hattendorf J., Schelling E., Zinsstag J. (2014). Prevalence of *Fasciola gigantica* infection in slaughtered animals in south-eastern Lake Chad area in relation to husbandry practices and seasonal water levels. BMC Vet. Res..

[174] Bisimwa N., Lugano R., Bwihangane B., Wasso S., Kinimi E., Banswe G., Bajope B. (2018). Prevalence of gastro-intestinal helminths in slaughtered cattle in Walungu territory, South Kivu Province, Eastern Democratic Republic of Congo. Austin J. Vet. Sci. Anim. Husb..

[175] Mochankana M.E., Robertson I.D. (2016). A retrospective study of the prevalence of bovine fasciolosis at major abattoirs in Botswana: Research communication. Onderstepoort J. Vet. Res..

[176] Mochankana M.E., Robertson I.D. (2018). Cross-sectional prevalence of *Fasciola gigantica* infections in beef cattle in Botswana. Trop. Anim. Health Prod..

[177] Chikowore T.J., Zishiri O.T., Mukaratirwa S. (2019). Phylogenetic analysis of *Fasciola* spp. isolated from slaughtered cattle in KwaZulu-Natal and Mpumalanga provinces of South Africa based on the cytochrome coxidase subunit I mitochondrial marker. Onderstepoort J. Vet. Res..

[178] Mucheka V.T., Lamb J.M., Pfukenyi D.M., Mukaratirwa S. (2015). DNA sequence analyses reveal co-occurrence of novel haplotypes of *Fasciola gigantica* with *F. hepatica* in South Africa and Zimbabwe. Vet. Parasitol..

[179] Van-Wyk C.I., Boomker J.D.F. (2011). Parasites of South African wildlife. XIX. The prevalence of helminths in some common antelopes, warthogs and a bushpig in the Limpopo province, South Africa. Onderstepoort J. Vet. Res..

[180] Alves R.M., Van Rensburg L., Van Wyk J.A. (1988). *Fasciola* in horses in the Republic of South Africa: A single natural case of *Fasciola hepatica* and the failure to infest ten horses either with *F. hepatica* or *Fasciola gigantica*. Onderstepoort J. Vet. Res..

[181] Gallivan G.J., Barker I.K., Culverwell J., Girdwood R. (1996). Prevalence of Hepatic Helminths and Associated Pathology in Impala (*Aepyceros melampus*) in Swaziland. J. Wildl. Dis..

[182] Nyirenda S.S., Sakala M., Moonde L., Kayesa E., Fandamu P., Banda F., Sinkala Y. (2019). Prevalence of bovine fascioliasis and economic impact associated with liver condemnation in abattoirs in Mongu district of Zambia. BMC Vet. Res..

[183] Phiri A.M., Chota A., Muma J.B., Munyeme M., Sikasunge C.S. (2011). Helminth parasites of the Kafue lechwe antelope (*Kobus leche kafuensis*): A potential source of infection to domestic animals in the Kafue wetlands of Zambia. J. Helminthol..

[184] Munyeme M., Munang’andu H.M., Muma J.B., Nambota A.M., Biffa D., Siamudaala V.M. (2010). Investigating effects of parasite infection on body condition of the Kafue lechwe (*Kobus leche kafuensis*) in the Kafue basin. BMC Res. Notes.

[185] Yabe J., Phiri I., Phiri A., Chembensofu M., Dorny P., Vercruysse J. (2008). Concurrent infections of *Fasciola*, *Schistosoma* and *Amphistomum* spp. in cattle from Kafue and Zambezi-river basins of Zambia. J. Helminthol..

[186] Phiri A.M., Phiri I.K., Sikasunge C., Chembensofu M., Monrad J. (2006). Comparative fluke burden and pathology in condemned and non-condemned cattle livers from selected abattoirs in Zambia. Onderstepoort J. Vet. Res..

[187] Phiri A.M., Phiri I.K., Monrad J. (2006). Prevalence of amphistomiasis and its association with *Fasciola gigantica* infections in Zambian cattle from communal grazing areas. J. Helminthol..

[188] Phiri A., Phiri I., Sikasunge C., Monrad J. (2005). Prevalence of fasciolosis in Zambian cattle observed at selected abattoirs with emphasis on age, sex and origin. J. Vet. Med..

[189] Phiri A.M., Phiri I.K., Siziya S., Sikasunge C.S., Chembensofu M., Monrad J. (2005). Seasonal pattern of bovine fasciolosis in the Kafue and Zambezi catchment areas of Zambia. Vet. Parasitol..

[190] Chauke E., Dhlamini Z., Mbanga J., Dube S. (2014). Characterization of *Fasciola gigantica* isolates from cattle from South-Western Zimbabwe using RAPD-PCR. IOSR J. Agric. Vet. Sci..

[191] Pfukenyi D.M., Mukaratirwa S. (2004). A retrospective study of the prevalence and seasonal variation of *Fasciola gigantica* in cattle slaughtered in the major abattoirs of Zimbabwe between 1990 and 1999. Onderstepoort J. Vet. Res..

[192] Chambers P. (1987). Carcase and offal condemnations at meat inspection in Zimbabwe. Zimb. Vet. J..

[193] Séré M., Sawadogo M.N., Pooda S.H., Kaboré B., Kaboré A., Tamboura H.H., Belem A.M.G. (2021). Diagnosis of fasciolosis in cattle slaughtered at the slaughterhouse of the urban commune of Dédougou in Burkina Faso. World J. Adv. Res. Rev..

[194] Periago M., Valero M., Panova M., Mas-Coma S. (2006). Phenotypic comparison of allopatric populations of *Fasciola hepatica* and *Fasciola gigantica* from European and African bovines using a computer image analysis system (CIAS). Parasitol. Res..

[195] Traoré S.I., Achi L.Y., Krauth S.J., Sanogo M., Zinsstag J., Utzinger J., N’Goran E.K. (2021). Distribution of bovine *Fasciola gigantica* (Cobbold, 1885) in the district des Savanes, northern Côte d’Ivoire. Geospat. Health..

[196] Kouadio J.N., Giovanoli Evack J., Achi L.Y., Fritsche D., Ouattara M., Silué K.D., Bonfoh B., Hattendorf J., Utzinger J., Zinsstag J. (2020). Prevalence and distribution of livestock schistosomiasis and fascioliasis in Côte d’Ivoire: Results from a cross-sectional survey. BMC Vet. Res..

[197] Addy F., Romig T., Wassermann M. (2018). Genetic characterisation of *Fasciola gigantica* from Ghana. Vet. Parasitol. Reg. Stud..

[198] Addy F., Gyan K., Arhin E., Wassermann M. (2020). Prevalence of bovine fasciolosis from the Bolgatanga abattoir, Ghana. Sci. Afr..

[199] Duedu K.O., Peprah E., Anim-Baidoo I., Ayeh-Kumi P.F. (2015). Prevalence of Intestinal Parasites and Association with Malnutrition at a Ghanaian Orphanage. Hum. Parasit. Dis..

[200] Tembely S., Galvin T., Craig T., Traore S. (1988). Liver fluke infections of cattle in Mali. An abattoir survey on prevalence and geographic distribution. Trop. Anim. Health Prod..

[201] Amor N., Farjallah S., Salem M., Lamine D.M., Merella P., Said K., Ben Slimane B. (2011). Molecular characterization of *Fasciola gigantica* from Mauritania based on mitochondrial and nuclear ribosomal DNA sequences. Exp. Parasitol..

[202] Binta B.-M., Kosisochukwu Dematus O., Peace A. (2021). Gastrointestinal Helminths Parasites of Cattle Slaughtered in Gwagwalada Abattoirs. SAJP.

[203] Iyiola O.A., Shaibu R.D., Shittu O., Alayande M.O., Rabiu M., Sulaiman M.K., Obarombi G.T. (2021). DNA barcode identification of a tropical liver fluke (*Fasciola gigantica*) in cattle from Ilorin, Northcentral Nigeria. Biologia.

[204] Adeoti O.M., Ajayi F., Olaoye O.J., Adedokun E.O., Adesina D.A., Adeoye K.A. (2020). Identification and prevalence of intestinal parasites from slaughtered cows in Saki and Ago-Are Abattoirs. World J. Adv. Res. Rev..

[205] Ahmad I., Yakubu Y., Chafe U.M., Bolajoko B.M., Muhammad U. (2020). Prevalence of fasciolosis (Liver flukes) infection in cattle in Zamfara, Nigeria: A slaughterhouse surveillance data utilizing postmortem examination. Vet. Parasitol. Reg. Stud. Rep..

[206] Aliyu A.A., Maikenti J., Aimankhu O., Ayuba S., Ahmed H., Haruna A., Idris A.M. (2020). Helminth Parasites of Goats and Sheep at Slaughterhouse in Lafia, Nasarawa State, Nigeria. Fudma J. Sci..

[207] Castilla Gómez de Agüero V., Luka J., Gandasegui J., Valderas-García E., Ajanusi O.J., Chiezey N.P., Martínez-Valladares M. (2020). *Fasciola hepatica* and *Fasciola gigantica* coexistence in domestic ruminants in Nigeria: Application of a PCR-based tool. Trop. Anim. Health Prod..

[208] Gimba U., Azare B., Airuoyuwa O. (2020). Occurrence of Gastrointestinal Helminthes Parasites of Cattle Slaughtered in Some Selected Abattoirs in Gwagwalada Area Council, Abuja. LAJANS.

[209] Ola-Fadunsin S.D., Ganiyu I.A., Rabiu M., Hussain K., Sanda I.M., Baba A.Y., Furo N.A., Balogun R.B. (2020). Helminth infections of great concern among cattle in Nigeria: Insight to its prevalence, species diversity, patterns of infections and risk factors. Vet. World..

[210] Ikenna-Ezeh N., Eke C., Ezeh I., Obi C., Chukwu C. (2019). Prevalence and hematological parameters of *Fasciola gigantica*-infected cattle in Nsukka, Southeastern Nigeria. Comp. Clin. Path..

[211] Iyiola O., Shittu O., Owolodun O., Animasaun D., Udeze A. (2018). Morphometric phenotypes and molecular identification of *Fasciola* species isolated from cattle in Ilorin, North-Central Nigeria. Sri Lankan J. Biol..

[212] Adewunmi O.A., Adekomi A.D., Akinseye Janet F., Okiki Pius A. (2017). Fascioliasis in Cattle Slaughtered for Consumption at Ado Ekiti Central Abattoir in Ekiti State, Nigeria. J. Biol. Agric. Healthcare.

[213] Afolabi O.J., Simon-Oke I.A., Ademiloye O. (2017). Gastro-intestinal parasites of bovine in Akure abattoirs, Nigeria. J. Entomol. Zool. Stud..

[214] Osinowo E.O., Adama J.Y., Popoola M.A., Shorinmade A.Y., Afolabi Q.O. (2017). Prevalence of *Fasciola gigantica* infestation in cattle slaughtered at Minna metropolitan abattoir in North Central Nigeria. J. Anim. Sci. Vet. Med..

[215] Adelabu D. (2016). Prevalence of *Fasciola gigantica* in slaughtered cattle in Ado municipal abattoir, Ado Ekiti, Ekiti state, Nigeria. J. Agric. For. Soc. Sci..

[216] Afolabi O.J., Olususi F.C. (2016). The prevalence of Fascioliasis among slaughtered cattle in Akure, Nigeria. Mol. Pathog..

[217] Elelu N., Ambali A., Coles G.C., Eisler M.C. (2016). Cross-sectional study of *Fasciola gigantica* and other trematode infections of cattle in Edu Local Government Area, Kwara State, north-central Nigeria. Parasites Vectors.

[218] Akoji O., Akpabio U., Ngulukun S. (2015). An Abattoir Study on the Prevalence of Fasciolosis in Cattle Slaughtered at Ubakala Slaughter House, Abia State, Nigeria. Eur. J. Appl. Sci..

[219] Abraham J., Jude I. (2014). Fascioliasis in cattle and goat slaughtered at Calabar abattoirs. J. Biol Agric. Healthcare.

[220] Adedipe O.D., Uwalaka E.C., Akinseye V.O., Adediran O.A., Cadmus S.I.B. (2014). Gastrointestinal helminths in slaughtered cattle in Ibadan, South-Western Nigeria. J. Vet. Med..

[221] Aliyu A.A., Ajogi I., Ajanusi O., Reuben R. (2014). Epidemiological studies of *Fasciola gigantica* in cattle in Zaria, Nigeria using coprology and serology. J. Public Health Epidemiol..

[222] Ngele K.K., Ibe E. (2014). Prevalence of fasciolopsis in cattle slaughtered at Eke market abattoir, Afikpo, Ebonyi state, Nigeria. Anim. Res. Int..

[223] Biu A., Paul B., Konto M., Ya’uba A. (2013). Cross sectional and phenotypic studies on fasciolosis in slaughter cattle in Maiduguri, Nigeria. J. Agric. Vet. Sci..

[224] Ejima I., Akor S. (2013). Fascioliasis in Cattle Slaughtered for Human Consumption in Minna Metropolis, Niger State, Nigeria?. J. Sci. Res..

[225] Odigie B., Odigie J. (2013). Fascioliasis in cattle: A survey of abattoirs in Egor, Ikpoba-Okha and Oredo Local Government Areas of Edo State, using histochemical techniques. Int. J. of Basic Appl. Innov. Research..

[226] Shitta K.B. (2013). Gastro-Intestinal Helminthes of Slaughtered Cattle at Wukari Abattoir Taraba State, North-Eastern Nigeria. Int. J. Epidemiol. Infect..

[227] Amadi A., Avoaja D., Essien E. (2012). Epidemiology of Helminth Parasites of West African Dwarf Goat (Capra Hircus) in Umuariaga in Ikwuano LGA, Abia State. J. Appl. Sci. Environ. Manag..

[228] Njoku-Tony R., Okoli G. (2011). Prevalence of fascioliasis among slaughter sheep in selected abattoirs in Imo state, Nigeria. J. Am. Sci..

[229] Ulayi B.M., Umaru-Sule B., Adamu S. (2007). Prevalence in *Dicrocoelium hospes* and *Fasciola gigantica* infections in cattle at slaughter in Zaria, Nigeria. J. Anim. Vet. Adv..

[230] Ekwunife C.A., Eneanya C.I. (2006). *Fasciola gigantica* in Onitsha and environs. Anim. Res. Int..

[231] Ngwu G.I., Ohaegbula A.B.O., Okafor F.C. (2004). Prevalence of *Fasciola gigantica*, *Cysticercus bovis* and some other disease conditions of cattle slaughtered in nsukka urban abattoir. Anim. Res. Int..

[232] Okoli I., Agoh E., Okoli G., Idemili G., Umesiobi D. (2000). Bovine and caprine fascioliasis in Enugu State, Nigeria: Retrospective analysis of abattoir records (1993-97) and six months prevalence study. Bull. Anim. Health Prod. Afr..

[233] Nwosu C.O., Srivastava G.C. (1993). Liver fluke infections in livestock in Borno State, Nigeria. Vet. Q..

[234] Njau B.C., Scholtens R.G., Kasali O. (1990). Parasites of sheep at the international Livestock Centre for Africa Debre Berhan station, Ethiopia. Prev. Vet. Med..

[235] Schillhorn van Veen T., Folaranmi D., Usman S., Ishaya T. (1980). Incidence of liver fluke infections (*Fasciola gigantica* and *Dicrocoelium hospes*) in ruminants in northern Nigeria. Trop. Anim. Health Prod..

[236] Ai L., Weng B., Elsheikhae H.M., Zhao G.H., Alasaadg S., Chend J.X., Lic J., Lic H.L., Wanga C.R., Chend M.X. (2011). Genetic diversity and relatedness of *Fasciola* spp. isolates from different hosts and geographic regions revealed by analysis of mitochondrial DNA sequences. Vet. Parasitol..

[237] Ali H., Ai L., Song H., Ali S., Lin R., Seyni B., Issa G., Zhu X. (2008). Genetic characterisation of *Fasciola* samples from different host species and geographical localities revealed the existence of *F. hepatica* and *F. gigantica* in Niger. Parasitol. Res..

[238] Mequaninit G., Mengesha A. (2021). Prevalence of Bovine Fasciolosis and Associated Economic Loss in Cattle Slaughtered at Kombolcha Industrial Abbatior. J. Vet. Med. Animal. Sci..

[239] Bekele D. (2019). The Prevalence and Economic Impact of Bovine Fasciolosis at Lalo Municipal Abattoir, Lalo Kile District, West Wollega, Ethiopia. Acta Parasitol..

[240] Bekele C.G., Tarekegn G.A. (2019). Major trematode infections of sheep in lemo woreda and associated economic loss due to liver condemnation at hossana town, southern Ethiopia. Int. J. Dev. Res..

[241] Desa G., Mitiku W. (2019). Prevalence and Economic Significance of Bovine Fasciolosis in Shambu Municipality Abattoir, Ethiopia. Acta Parasitol..

[242] Zewde A., Bayu Y., Wondimu A. (2019). Prevalence of bovine fasciolosis and its economic loss due to liver condemnation at Wolaita Sodo Municipal Abattair, Ethiopia. Vet. Med. Int..

[243] Getnet A., Bayih T. (2018). Prevalence of Bovine Fasciolosis and Economic Importance in Wulnchit Municipal Abatoir, Ethiopia. Glob. J. Sci. Front. Res..

[244] Hayider N., Mekuria S., Mekibib B. (2018). Major trematodes of cattle slaughtered at Hirna municipal Abattoir: Prevalence, associated risk factors and test agreement of sedimentation technique in Ethiopia. J. Parasitol. Vector Biol..

[245] Kibeb L., Hagos A. (2018). Equine fasciolosis, a growing problem in Arsi-Bale highlands of Oromia region, Southeastern Ethiopia. Int. J. Fauna Biol..

[246] Meharenet B. (2018). Prevalence of Bovine fasciolosis and economic significance in and around Chora Wereda, Western Ethiopia. Acta Parasitol..

[247] Metages Y., Mathewos M., Ademasu A., Mikias E. (2018). Abatoir Survey on Prevalence and Economic Significance of Fasciolosis in Small Ruminants Slaughtered in Addis Ababa Municipal Abatoir, Ethiopia. Acta Parasitol..

[248] Mohammadnur M., Geleta M. (2018). Prevalence of Bovine Fasciolosis and its Economic Significance at Robe Municipal Abattoir. Glob. J. Comput. Sci. Technol..

[249] Alemayehu G., Aynalem Y., Haile A. (2017). Prevalence of Bovine fasciolosis infection in Hossana municipal abattoir, Southern Ethiopia. J. Nat. Sci. Res..

[250] Amsalu T. (2017). Prevalence of ovine fasciolosis and loss due to liver condemnation at Bahir Dar Town, Ethiopia. Adv. Biol. Res..

[251] Berhe N., Tefera Y., Tintagu T., Muleta W. (2017). Small Ruminant Fasciolosis and its Direct Financial Loss in Dessie Municipal Abattoir Northeastern Ethiopia. J. Vet. Sci. Technol..

[252] Eshetu E., Thomas N., Awukew A., Goa A., Butako B. (2017). Study on the Prevalence of Bovine Fasciolosis and Estimated Financial Losses Due to Liver Condemnation: In case of Angacha Woreda, Kambata Tembaro Zone, Southern Ethiopia. J. Biol Agric. Healthcare.

[253] Kassye D., Gebeyehu M., Mekonnen D. (2017). Prevalence and Associated Risk Factors of Small Ruminant Fasciolosis in Haramaya District, Eastern Ethiopia. Acta Parasitol..

[254] Meshesha M., Tesfaye W. (2017). Prevalence of fasciolosis in cattle slathered at Hosanna Municipal Abattoir, Southern Ethiopia. Int. J. Adv. Res. Biol. Sci..

[255] Oyda S., Sheferaw D., Aragaw K. (2017). *Fasciola* infection prevalence and financial loss due to liver condemnation in cattle slaughtered at Wolaita Sodo municipal abattoir, southern Ethiopia. Sci. J. Vet. Adv..

[256] Tesfaye W., Tigist T. (2017). Incidence and economic impact of fasciolosis in Wolkite town, Community Abattoir. J. Vet. Med. Anim. Health..

[257] Worku T., Herago T., Amajo M. (2017). Prevalence and Financial Losses Associated with Bovine Fasciolosis at Asella Municipal Abattoir, South Eastern Ethiopia. Adv. Biol. Res..

[258] Yasin H., Tsegay T., Niraj K. (2017). Prevalence of Bovine Trematodes and Associated Risk Factors at Abergelle Export Abattoir, Mekelle, Tigray, Ethiopia. Ethiop. J. Vet. Sci. Anim. Prod..

[259] Abebe W., Keffale M., Adugna S. (2016). Prevalence of bovine fasciolosis and its economic significance in Bonga Abattoir, Kafa Zone, Southwestern Ethiopia. Acta Parasitol..

[260] Ayele M., Ayele B., Belete A. (2016). Prevalence and associated risk factors of helminth parasites of small ruminants slaughtered at HELIMEX abattoir, Ethiopia. J. Vet. Med. Anim. Health.

[261] Tengase D., Kebede A., Taqaba E. (2016). Comparison on sensitivity of coprological and post mortem examination in diagnosis of bovine fasciolosis: Prevalence of the disease in Bedele municipal abattoir. Afr. J. Basic Appl. Sci..

[262] Yitayal G., Taddie W. (2020). Prevalence Bovine Fasciolosis in Postmortem Examination at Bahir Dar Municipal Abattoir, Bahir Dar, Ethiopia. J. Anim. Husb. Dairy Sci..

[263] Yusuf M., Nuraddis I., Tafese W., Deneke Y. (2016). Prevalence of bovine fasciolosis in municipal abattoir of Haramaya, Ethiopia. Food Sci. Qual. Manag..

[264] Alemu A., Abebe B. (2015). Fasciolosis: Prevalence, evaluation of flotation and simple sedimentation diagnostic techniques and monetary loss due to liver condemnation in cattle slaughtered at Wolaita Soddo municipal abattoir, Southern Ethiopia. Food Sci. Qual. Manag..

[265] Assefa A., Assefa Z., Beyene D., Desissa F. (2015). Prevalence of bovine fasciolosis in and around inchini town, west showa zone, Adaa Bega Woreda, Centeral Ethiopia. J. Vet. Med. Anim. Health.

[266] Belina D., Demissie T., Ashenafi H., Tadesse A. (2015). Comparative pathological study of liver fluke infection in ruminants. Indian J. Vet. Pathol..

[267] Geresu M.A., Hailemariam Z., Mamo G., Tafa M., Megersa M. (2015). Prevalence and associated risk factors of major gastrointestinal parasites of pig slaughtered at Addis Ababa Abattoirs Enterprise, Ethiopia. J. Vet. Sci. Technol..

[268] Girmay T., Teshome Z., Hailemikael A. (2015). Prevalence and Economic Losses of bovine fasciolosis at Hawzien Abattoir, Tigray Region Northern Ethiopia. J. Vet. Adv..

[269] Legesse D.T. (2015). Comparison of Two Parasitological Tests and Post Mortem Examination to Detect Prevalence of Bovine Fasciolosis at Ambo Town Municipal Abattoir, West Shewa Zone, Ethiopia. Open Access Libr..

[270] Moje N., Mathewos S., Desissa F., Regassa A. (2015). Cross-sectional study on bovine fasciolosis: Prevalence, coprological, abattoir survey and financial loss due to liver condemnation at Areka Municipal Abattoir, Southern Ethiopia. J. Vet. Med. Anim. Health..

[271] Tsegaye B., Gebeyehu B. (2015). Fasciolosis: Abattoir prevalence and severity of liver lesions in sheep slaughtered at Debre-Birhan Municipal Abattoir, North East Ethiopia. Int. J. Agric. Sci. Res..

[272] Yitagezu A., Tefera W., Mahendra P. (2015). Prevalence of bovine fasciolosis and its economic impact in Bedele, Ethiopia. Haryana Vet..

[273] Asrese N.M., Ali M.G. (2014). Bovine Fasciolosis: Prevalence and Economic Significance in Southern Ethiopia. Acta Parasitol..

[274] Chakiso B., Menkir S., Desta M. (2014). On farm study of bovine fasciolosis in Lemo district and its economic loss due to liver condemnation at Hossana municipal abattoir, southern Ethiopia. Int. J. Curr. Microbiol. Appl. Sci..

[275] Fetene A., Addis M. (2014). An abattoir survey on the prevalence and monetary loss of fasciolosis among cattle slaughtered at Dangila municipal abattoir, Ethiopia. J. Vet. Med. Anim. Health..

[276] Sisay A., Takele B., Menda S. (2014). Prevalence of bovine fasciolosis and economic loss due to liver condemnation at Debre Markos municipal abattoir, Northern Ethiopia. Sci. J. Vet. Adv..

[277] Zeleke M.A., Gurmesa M., Tesfaye T. (2014). Economic significance of fasciolosis at Mettu municipal abattoir, southwest Ethiopia. J. Adv. Vet. Res..

[278] Aregay F., Bekele J., Ferede Y., Hailemelekot M. (2013). Study on the prevalence of bovine fasciolosis in and around Bahir Dar, Ethiopia. Ethiop. Vet. J..

[279] Kebede W., Pal M., Deressa A., Ritwick R. (2013). Prevalence and economic significance of fasciolosis in cattle slaughtered at Elfora abattoir, Gondar. J. Nat. Hist..

[280] Petros A., Kebede A., Wolde A. (2013). Prevalence and economic significance of bovine fasciolosis in Nekemte municipal abattoir. J. Vet. Med. Anim. Health..

[281] Zeleke M.A., Tadesse A., Kumar B.A. (2013). Epidemiology of fasciolosis in Southwest Ethiopia. J. Adv. Vet. Res..

[282] Zewdneh T., Ekwal I., Tsegabirhan K., Yohannes T., Kidane W. (2013). Prevalence of gastrointestinal parasites and *Cryptosporidium* species in extensively managed pigs in Mekelle and urban areas of southern zone of Tigray region, Northern Ethiopia. Vet. World..

[283] Aragaw K., Negus Y., Denbarga Y., Sheferaw D. (2012). Fasciolosis in slaughtered cattle in Addis Ababa abattoir, Ethiopia. Glob. Vet..

[284] Belay E., Molla W., Amare A. (2012). Prevalence and economic losses of bovine fasciolosis in dessie Municipal Abattoir, South Wollo Zone, Ethiopia. Eur. J. Biol. Sci..

[285] Chanie M., Begashaw S. (2012). Assessment of the Economic Impact and Prevalence of Ovine Fasciolosis in Menz Lalo Midir District, Northeast Ethiopia. Vet. World.

[286] Demssie A., Birku F., Biadglign A., Misganaw M., Besir M., Addis M. (2012). An Abattoir survey on the prevalence and monetary loss of fasciolosis in cattle in Jimma Town, Ethiopia. Glob. Vet..

[287] Gebrecherkos B.A. (2012). Prevalence of bovine fascilosis in municipal Abbatoir of Adigrat, Tigray, Ethiopia. Rev. Electron. Vet..

[288] Kedir S., Deressa B., Tigre W. (2012). Small Ruminant Fasciolosis in Jimma Area of Southwestern Ethiopia: Its Epidemiology and Minimum Monetary Loss. Glob. Vet..

[289] Messele E.Y., Gashaw A., Pal M., Girmay G. (2012). Prevalence of bovine fasciolosis, amplitude of liver condemnation and its economic impact in Municipal Abattoir of Mekelle, Ethiopia. Int. J. Livest. Res..

[290] Mulat N., Basaznew B., Mersha C., Achenef M., Tewodros F. (2012). Comparison of coprological and postmoretem examinations techniques for the deterimination of prevalence and economic significance of bovine fasciolosis. J. Adv. Vet. Res..

[291] Regassa A., Woldemariam T., Demisie S., Moje N., Ayana D., Abunna F. (2012). Bovine fasciolosis, Coprological, Abattoir survey and financial loss due to liver condemnation in Bishooftu Municipal Abattoir, Central Ethiopia. Eur. J. Biol. Sci..

[292] Tesfay H., Dejene T., Kebede E. (2012). Prevalence of bovine fasciolosis and its associated risk factors in Mekelle municipal abattoir. J. Drylands.

[293] Dagnachew S., Amamute A., Temesgen W. (2011). Epidemiology of gastrointestinal helminthiasis of small ruminants in selected sites of North Gondar zone, Northwest Ethiopia. Ethiop. Vet. J..

[294] Kifle D., Hiko A. (2011). Abattoir survey on the prevalence and monitory loss associated with Fasciolosis in sheep and goats. Int. J. Livest. Prod..

[295] Mulugeta S. (2011). Prevalence of Bovine Fasciolosis and its Economic Significance in and Around Assela, Ethiopia Shiferaw Mulugeta, Feyisa Begna, Ephrem Tsegaye. Glob. J. Med. Res..

[296] Abunna F., Asfaw L., Megersa B., Regassa A. (2010). Bovine fasciolosis: Coprological, abattoir survey and its economic impact due to liver condemnation at Soddo municipal abattoir, Southern Ethiopia. Trop. Anim. Health Prod..

[297] Bekele M., Getachew Y. (2010). Bovine Fasciolosis: Prevalence and its economic loss due to liver condemnation at Adwa Municipal Abattoir, North Ethiopia. Ethiop. J. Appl. Sci. Technol..

[298] Berhe G., Tadesse G., Kiros H., Abebe N. (2010). Concurrent infection of hydatidosis and fasciolosis in cattle slaughtered at Mekelle municipal abattoir, Tigray Region. Ethiop. Vet. J..

[299] Getachew M., Innocent G., Trawford A., Reid S., Love S. (2010). Epidemiological features of fasciolosis in working donkeys in Ethiopia. Vet. Parasitol..

[300] Getachew M., Trawford A., Feseha G., Reid S. (2010). Gastrointestinal parasites of working donkeys of Ethiopia. Trop. Anim. Health Prod..

[301] Ibrahim N., Wasihun P., Tolosa T. (2009). Prevalence of Bovine Fasciolosis and Economic Importance Due to Liver Condemnation At Kombolcha Industrial Abattoir, Ethiopia. IJVM.

[302] Sissay M.M., Uggla A., Waller P.J. (2007). Prevalence and seasonal incidence of nematode parasites and fluke infections of sheep and goats in eastern Ethiopia. Trop. Anim. Health Prod..

[303] Yilma J., Mesfin A. (2000). Dry season bovine fasciolosis in Northwestern part of Ethiopia. Rev. Med. Vet..

[304] Bekele T., Kasali O.B., Woldemariam W. (1992). Endoparasite prevalences of the highland sheep in Ethiopia. Prev. Vet. Med..

[305] Kithuka J.M., Maingi N., Njeruh F.M., Ombui J.N. (2002). The prevalence and economic importance of bovine fasciolosis in Kenya--an analysis of abattoir data. Onderstepoort J. Vet. Res..

[306] Mzembe S., Chaudhry M. (1981). The epidemiology of fascioliasis in Malawi part II. Epidemiology in the definitive host. Trop. Anim. Health Prod..

[307] Nzalawahe J., Kassuku A.A., Stothard J.R., Coles G.C., Eisler M.C. (2015). Associations between trematode infections in cattle and freshwater snails in highland and lowland areas of Iringa Rural District, Tanzania. Parasitology.

[308] Kamwela K., Kassuku A., Nonga H. (2013). Prevalence and financial losses associated with bovine fasciolosis at SAAFI and Sumbawanga municipal abattoirs, Rukwa, Tanzania. Tanzan. Vet. J..

[309] Nzalawahe J., Komba E.V. (2013). Occurrence and seasonal predisposition of fasciolosis in cattle and goats slaughtered in Kasulu District abattoir, Western Tanzania. Res. Opin. Anim. Vet. Sci..

[310] Mhoma J., Kanyari P., Kagira J. (2011). The prevalence of gastrointestinal parasites in goats in urban and peri-urban areas of Mwanza City, Tanzania. Sci. Parasitol..

[311] Mwabonimana M., Kassuku A., Ngowi H., Mellau L., Nonga H., Karimuribo E. (2009). Prevalence and economic significance of bovine fasciolosis in slaughtered cattle at Arusha abattoir, Tanzania. Tanzan. Vet. J..

[312] Nonga H., Mwabonimana M., Ngowi H., Mellau L., Karimuribo E. (2009). A retrospective survey of liver fasciolosis and stilesiosis in livestock based on abattoir data in Arusha, Tanzania. Trop. Anim. Health Prod..

[313] Keyyu J., Kassuku A.A., Msalilwa L., Monrad J., Kyvsgaard N. (2006). Cross-sectional prevalence of helminth infections in cattle on traditional, small-scale and large-scale dairy farms in Iringa district, Tanzania. Vet. Res. Commun..

[314] Keyyu J., Monrad J., Kyvsgaard N., Kassuku A. (2005). Epidemiology of *Fasciola gigantica* and amphistomes in cattle on traditional, small-scale dairy and large-scale dairy farms in the southern highlands of Tanzania. Trop. Anim. Health Prod..

[315] Boorder J.D. (1992). Spread of *F. gigantica* in Tanzania. Vet. Rec..

[316] Choi Y.J., Fontenla S., Fischer P.U., Le T.H., Costábile A., Blair D., Brindley P.J., Tort J.F., Cabada M.M., Mitreva M. (2020). Adaptive radiation of the flukes of the family Fasciolidae inferred from genome-wide comparisons of key species. Mol. Biol. Evol..

[317] Nambafu J., Musisi J.S., Mwambi B., James K., Patrick O., Joel B., Herbert I., Iramiot J.S. (2015). Prevalence and economic impact of bovine fasciolosis at Kampala City abattoir, Central Uganda. Br. Microbiol. Res. J..

