# Supplementary material for: Potential Hybridization of Fasciola hepatica and F. gigantica in Africa—A Scoping Review
Source: Pathogens. 2022 Nov 6;11(11):1303. doi: 10.3390/pathogens11111303 (PMC9695073; doi:10.3390/pathogens11111303)
Supplement: Supplementary file 1 [file pathogens-11-01303-s001.zip › pathogens-1995197-supplementary.pdf]

Table S1. Summary of African studies reporting on the presence of *Fasciola* species recovered from various definitive hosts.

Author	Aim/ objective	Country	*F. hepatica*	*F. gigantica*	Both	Host	Diagnostic tool
[1]	Identify the phenotypic features and genetic characterization of adult fasciolids infecting buffaloes in Aswan, Egypt.	Egypt			✓	Buffalo, cattle, sheep	Morphology, molecular
[2]	Molecularly characterise *Fasciola* flukes using the ITS-1 & 2 nuclear markers to confirm species and any hybrid forms.	Chad		✓		Cattle, sheep, goats	Molecular
[5]	Determine the prevalence of fasciolosis and costs incurred due to liver condemnation and evaluate the sensitivity of direct sedimentation method for diagnosis of fasciolosis in cattle slaughtered at Hawassa Municipal abattoir.	Ethiopia			✓	Cattle	Morphology
[29]	Molecularly ascertain the nature of *Fasciola* population derived from different hosts and different geographic locations in Egypt.	Egypt		✓		Cattle, buffalo, sheep	Molecular
[32]	Morphological and molecular characterization of *Fasciola* spp. collected from cattle slaughtered at abattoirs located in the two provinces of South Africa, where two species are endemic.	South Africa			✓	Cattle	Morphology, molecular
[38]	Investigate *Fasciola* species in cattle and lymnaeid snails in Dakhla Oasis, El-Wadi El-Gadid, Egypt. Gross and microscopic findings were confirmed by duplex PCR and sequence analysis of the resulting mitochondrial cox1 amplicons.	Egypt	✓			Cattle	Morphology, molecular
[40]	Identify *Fasciola* spp. infection in cattle and investigate its molluscan intermediate host with mitochondrial DNA-targeting PCR assay in Dakhla Oasis, El wadi Elgadid, Egypt.	Egypt	✓			Cattle	Molecular
[53]	Determine the epidemiology of fasciolasis in Gafsa oases.	Tunisia	✓			Cattle, human	Morphology, serology
[78]	Study the availability of freshwater snails and their infection rates with larval trematodes in the Kafue wetlands and to assess the level of trematode infections in cattle accessing the snail-infested areas during a period of highest water contact.	Zambia		✓		Cattle	Morphology

[80]	Determine the epidemiology of *F. gigantica* in cattle in the highveld and lowveld communal grazing areas of Zimbabwe, and to use this information to recommend appropriate measures to control the parasite.	Zimbabwe		✓		Cattle	Morphology
[98]	Observations were made on a ranch in the Central Rift Valley of Kenya where fasciolosis was endemic.	Kenya		✓		Cattle	Morphology
[100]	Investigate the occurrence of fasciolosis in bovids at lowand high altitude areas on the slopes of Mount Elgonand also assessed animal condition (i.e., body signalment).	Uganda		✓		Cattle	Morphology
[102]	Investigate the epidemiology of trematode infections in cattle under different cattle management systems in order to generate base line data that will provide information for the design of effective control strategies.	Tanzania				Cattle	Morphology
[105]	Differentiate between the three fasciolid worms encountered in sheep and cattle in Sohag through a simple and rapid PCR-restriction fragment length polymorphism (RFLP) assay, using the common restriction enzymes AvaII based on a 618-bp-long sequence of the 28S rRNA gene.	Egypt			✓	Sheep, cattle	Morphology, molecular
[106]	Determine the occurrence rate of *Fasciola* spp. in sheep as measured by postmortem examination of slaughtered animals at abattoirs.	Egypt			✓	Buffalo	Morphology, molecular
[107]	To determine the prevalence of fascioliasis in cattle, and to describe the histopathological changes in liver and lung.	Egypt			✓	Cattle	Morphology
[108]	Use sequence analysis of the ITS-2 region of rDNA and a highly repetitive DNA sequences to determine the identity and heterogeneity among *Fasciola* isolated from buffalo, cow, and sheep hosts.	Egypt		✓		Cattle, buffalo	Molecular
[109]	DNA multi-marker characterization of the first finding of *F. gigantica* in sheep in Algeria by the complete sequences of rDNA ITS-1 and ITS-2 and mtDNA cox1 and nad1 genes.	Algeria		✓		Sheep	Molecular
[140]	*Fasciola* isolates from Algeria were identified at the species level by using the nuclear pepck and pold markers.	Algeria	✓			Cattle	Molecular

[141]	Conduct an epidemiological in the slaughterhouse investigation to update the knowledge on these two flukes.	Algeria	✓			Cattle	Morphology
[142]	Characterise *Fasciola* flukes from several populations located within the Tunisian-Algerian border, and to test if there is evidence for gene flow between these populations.	Algeria, Tunisia	✓			Cattle	Molecular
[143]	Assessed its potential to diagnose *Fasciola hepatica* infection. Also determined the prevalence of fasciolosis in slaughtered cattle of the Mitidja area and evaluate different risk factors: sex, age, and season.	Algeria	✓			Cattle	Morphology, serology
[144]	Investigated the genetic variability of *Fasciola* samples, recovered from different Algerian provinces, based on several molecular markers (ITS, COI-trnT-rrnL, NADI, and COI).	Algeria	✓			Cattle	Morphology, molecular
[145]	Determine the prevalence of fascioliasis in cattle, sheep and, for the first time, in goats and dromedary in two different regions: El 182 Tarf (Humid climate), northeastern Algeria and Ouargla (Saharan climate), south Algeria economic losses were evaluated.	Algeria	✓			Cattle, sheep, goat, camel	Morphology
[146]	Establish the presence of *Fasciola hepatica* on farms in north-eastern Algeria.	Algeria	✓			Cattle	Morphology
[147]	Characterise *Fasciola* spp. samples from Tunisia and Algeria from different definitive host animals and geographical localities by sequencing the region spanning the Internal Transcribed Spacers (ITS)1, the 5.8S and the ITS2 of nuclear ribosomal DNA (rDNA) and the mitochondrial Cytochrome c Oxidase I (COI) gene.	Algeria, Tunisia	✓			Cattle, sheep	Molecular
[148]	Clarify the prevalence of *Fasciola* species and associated risk factors of infection among cattle and goats.	Egypt			✓	Cattle, goat	Molecular
[149]	Determine the current prevalence of cattle fascioliasis at abattoirs in El-Kharga city, New Valley Governorate, Egypt, and investigate the changes in serum biochemical and immunological parameters and oxidative stress factors due to	Egypt	✓			Cattle	Morphology

	Fasciola spp. infection in terms of meat quality and immune response.						
[150]	Estimate the prevalence of *F. hepatica* infection in water buffalo in three governorates of the Nile Delta in Egypt and identify the underlying risk factors associated with the infection.	Egypt	✓			Buffalo	Morphology
[151]	The nad1 and cox1 sequences of Nigerian *Fasciola* flukes were phylogenetically compared with those from Egypt and Zambia (unpublished data) to elucidate the expansion history of *Fasciola* flukes within the African continent.	Nigeria		✓		Cattle	Molecular
[152]	Perform molecular characterization of *Fasciola* isolates from human and sheep in Assiut, Upper Egypt by sequence and phylogenetic analyses of ITS-1 and ITS-2 of ribosomal DNA genes.	Egypt	✓			Sheep	Molecular
[153]	Use polymerase chain reaction (PCR) for the description of *Fasciola* sp. in cattle and sheep and comparative study of genetic construction of *Fasciola* spp. in them.	Egypt			✓	Cattle, sheep	Morphology, molecular
[154]	Determine occurrence of *Fasciola* spp. in donkeys at two localities in Egypt (Giza Zoo and Zagazig region), and to identify the flukes to the species level using PCR-RFLP of amplified 28S rRNA gene for the first time.	Egypt		✓		Donkey	Morphology, molecular
[155]	Spotlight on speciation of *Fasciola* population in Egypt.	Egypt			✓	Cattle, sheep, buffalo	Molecular
[156]	Surveying the species and natural prevalence of helminths infecting sheep as well as seasonal fluctuation in different locations in Beni-Suef province, Egypt.	Egypt		✓		Sheep	Morphology
[157]	Identification and differentiation of the two species of *Fasciola* (*F. gigantica* and *F. hepatica*) for epidemiological applications especially in regions where the two species overlap in Egypt.	Egypt			✓	Cattle, sheep	Morphology, molecular
[158]	Presents evidence for the existence of four well-separated 30 clades: African *F. gigantica*-like flukes, Indian *F. gigantica*-like	Egypt, Tanzania			✓	-	Morphology, molecular

	flukes, European *F. hepatica* like flukes and African high-altitude *F. hepatica*-like flukes.						
[159]	Used microsatellite markers previously described for *F. hepatica* (Hurtrez-Bousses et al., 2004) to analyze Egyptian *Fasciola* population utilizing reference samples of both fluke species.	Egypt			✓	Cattle, buffalo, sheep	Molecular
[160]	Investigate the development of miracidium and hatching mechanism of *Fasciola* eggs.	Egypt			✓	-	Morphology
[161]	Identify and describe the liver fluke (*Fasciola*) parasitizing buffaloes, cows and sheep in Qena Governorate, Egypt. Also renewing the present information on the prevalence and worm burden per liver.	Egypt			✓	Cattle, buffalo, sheep	Morphology
[162]	Validate the identification of *Fasciola* species based on the shape and size of eggs shed by humans, characterizing their morphometric traits using a computer image analysis system (CIAS).	Egypt			✓	Cattle, human	Morphology
[163]	Identification of the Egyptian species of *Fasciola* depending on morphologic, morpho anatomic and morphometric differences.	Egypt			✓	Cattle, buffalo	Isoelectric focusing, morphology
[164]	Assess the prevalence of *F. gigantica* and *C. tenuicollis [Taenia hydatigena].*	Egypt		✓		Sheep, goat	Morphology
[165]	Examined donkey livers to reveal *Fasciola hepatica* infection.	Morocco	✓			Donkey	Morphology
[166]	Determine the prevalence of the disease and estimate the monetary losses due to livers condemnation among indigenous Nilotic and exotic Ankole cattle in Juba main Slaughterhouse, South Sudan.	South Sudan		✓		Cattle	Morphology
[167]	Use genomic DNA from confirmed specimens of adult *Fasciola hepatica* and *Fasciola gigantica* to develop and validate a robust, PCR-based method for distinguishing these two species, using primers based on RAPD-derived sequences.	Ghana, Sudan		✓		Cattle	Morphology, molecular

[168]	Investigate the prevalence of caprine fasciolosis and the epidemiological aspects.	Sudan		✓		Goat	Morphology
[169]	Highlight the important parasitical diseases could be excreted by goats and recontaminate the pasture and the factors that may affect the transmission of these parasitical disease.	Sudan		✓		Goat	Morphology
[170]	Characterize *Fasciola* spp. samples parasitizing horses from Tunisia by sequences of the 1st and 2nd internal transcribed spacers (ITS-1 and ITS-2) of ribosomal DNA (rDNA).	Tunisia	✓			Horse	Molecular
[171]	Evaluate the prevalence and infection intensity of the main hepatic and rumenal distomes of cattle in Vina Division (Adamawa - Cameroon) with the aim of contributing to the development of effective strategies to fight against distomatoses in Cameroon.	Cameroon		✓		Cattle	Morphology
[172]	Determine the *Fasciola* species infecting cattle, develop a species-specific serum antibody ELISA, assess the performance of the ELISA, and use it to assess the prevalence of *F. gigantica* exposure in two important cattle-rearing areas of Cameroon.	Cameroon		✓		Cattle	Molecular
[173]	Investigate the mobile pastoralists' priority concern of fasciolosis in their livestock.	Chad		✓		Cattle, sheep, goat	Morphology
[174]	Determine the prevalence of various gastrointestinal helminth parasitic infections in slaughtered cattle.	Democratic Republic of Congo		✓		Cattle	Morphology
[175]	Determine the prevalence of *Fasciola gigantica* infections in slaughtered cattle based on the records from the two main export abattoirs in Botswana for the period 2001–2010.	Botswana		✓		Cattle	-
[176]	Determine the prevalence and distribution of *F. gigantica* in cattle in Botswana.	Botswana		✓		Cattle	Morphology
[177]	Identifying *Fasciola* spp. isolates collected from cattle slaughtered at abattoirs located in KwaZulu-Natal (KZN) and Mpumalanga (MP) provinces using the (mtDNA) COI region	South Africa			✓	Cattle	Molecular

	and examining interspecies genetic diversity among the isolates using COI haplotypes.						
[178]	Use phylogenetic analyses based on nuclear ribosomal ITS1-5.8S-ITS2 (ITS) and mitochondrial cytochrome oxidase 1 (CO1) sequences to identify *Fasciola* spp. infecting cattle in Zimbabwe and selected locations in the KwaZulu-Natal and Mpumalanga provinces of South Africa and to determine their genetic diversity and structure.	Zimbabwe, South Africa			✓	Cattle, antelope, eland, duiker	Molecular
[179]	Determine the kinds and numbers of helminth that occur in different wildlife hosts in the area as well as whether any zoonotic helminths were present.	South Africa	✓			Kudu	Morphology
[180]	Obtain an indication of the role horses could play as reservoirs of infestation in the epidemiology of fascioliasis in this country.	South Africa	✓			Horse	Morphology
[181]	Report the patterns of prevalence and lesions associated with hepatic helminthiasis in impala in the Mlawula-Mbuluzi-Simunye Nature Reserve and Protected Area complex in northeastern Swaziland.	Swaziland		✓		Impala	Morphology
[182]	Estimate the prevalence and evaluate the direct economic loss caused by the *Fasciola* spp. in slaughtered cattle in a five-year period.	Zambia			✓	Cattle	Morphology
[183]	Evaluate the presence and distribution of helminths of lechwe and to compare these infection levels in lechwe of the Lochinvar and Blue Lagoon national parks.	Zambia		✓		Antelope	Morphology
[184]	Investigated the effect of the ecto and endo-parasites found in the Kafue basin on body condition of free ranging Kafue lechwe, as a way of examining the role of parasite infestation on reducing the Kafue lechwe population.	Zambia		✓		Kafue Lechwe	Morphology
[185]	Determine the occurrence and extent of concurrent *F. gigantica*, *Schistosoma* spp. and *Amphistomum* spp. infections in free-ranging cattle in Zambia, and to investigate their interactions.	Zambia		✓		Cattle	Morphology

[186]	Determine the liver fluke burden and pathology in condemned and non-condemned cattle livers from selected abattoirs in Zambia, and to determine whether there was any correlation between fluke burden and faecal fluke egg count.	Zambia		✓		Cattle	Morphology
[187]	Determine the prevalence and distribution of amphistomiasis in cattle in the three major cattle keeping areas of Zambia using coprological examination; and its association with *F. gigantica* infection as well as examining the associations of origin, sex and age on its occurrence.	Zambia		✓		Cattle	Morphology
[188]	Determine the prevalence and some factors influencing occurrence of fasciolosis in cattle, carried out at selected major abattoirs in Zambia.	Zambia		✓		Cattle	Morphology
[189]	Determine the seasonality of *F. gigantica* in cattle in the Kafue and Zambezi catchment areas of Southern province of Zambia.	Zambia		✓		Cattle	Morphology
[190]	Characterize *Fasciola gigantica* isolates from cattle in different localities using RAPD-PCR.	Zimbabwe		✓		Cattle	Molecular, morphology
[191]	Determine the prevalence and seasonal variation of *F. gigantica* in cattle, based on records of the major abattoirs in Zimbabwe during the period of 1990–1999.	Zimbabwe		✓		Cattle	-
[192]	Carcass and offal condemnations at meat inspection in Zimbabwe	Zimbabwe	✓			Cattle	Morphology
[193]	Identify the responsible fluke species and estimate the prevalence, the risk factors and the economic losses associated with this pathology among the butchers of the commune.	Burkina Faso		✓		Cattle	Morphology
[194]	Characterize the morphometry of the adult stage of *F. gigantica* from bovines in natural liver fluke populations from Burkina Faso by means of computer image analysis and the use of an allometric mode.	Burkina Faso		✓		-	Morphology

[195]	Deepen the understanding of the distribution of fascioliasis in the entire district des Savanes, which is the main region for cattle husbandry in Côte d'Ivoire.	Côte d'Ivoire		✓		Cattle, sheep	Morphology
[196]	Provide an overview of the epidemiology of schistosomiasis and fascioliasis in livestock by investigating the prevalence and the distribution of these diseases across Côte d'Ivoire.	Côte d'Ivoire		✓		Cattle, sheep, goats	Morphology
[197]	Used molecular genetic tools to characterise the intraspecific variations within *Fasciola* spp. in cattle from the northernmost parts (Bolgatanga and Wa) of Ghana and show how Ghanaian *Fasciola* spp. are genetically related to haplotypes from other parts of Africa.	Ghana		✓		Cattle	Molecular
[198]	Determine the fasciolosis situation in terms of prevalence, distribution, and identification of the *Fasciola* species in cattle in Ghana's Upper East Region.	Ghana		✓		Cattle	Molecular
[199]	Identify the prevalence of intestinal parasites and their associations with malnutrition in one of the major orphanages run by the Department of Social Welfare in Accra, Ghana.	Ghana	✓			Human	Morphology
[200]	Six hundred and ninety-two cattle slaughtered in various abattoirs in Mali between August 1983 and November 1984 were examined for evidence of *Fasciola gigantica* and *Dicrocoelium hospes* injections.	Mali		✓		Cattle	-
[201]	Describe for the first time the molecular characterization of *F. gigantica* from different definitive hosts and geographical localities of Mauritania.	Mauritania		✓		Cattle, sheep	Molecular
[202]	Determine the prevalence of gastrointestinal helminth parasites in cattle slaughtered in Gwagwalada abattoirs.	Nigeria		✓		Cattle	Morphology
[203]	Explored the use of DNA barcoding approach as a reliable molecular tool for precise species identification and molecular differentiation of *F. gigantica* samples.	Nigeria		✓		Cattle	Morphology, molecular
[204]	Provided valuable information on the burden of parasite among cattle in Saki, Oyo State.	Nigeria			✓	Cattle	Morphology

[205]	Determine the occurrence of bovine fasciolosis, and correlate determinants of infection in cattle slaughtered at Zamfara municipal abattoir between 2016 and 2017.	Nigeria		✓		Cattle	Morphology
[206]	Determine helminth parasites of goats and sheep at slaughter in Lafia from June to July 2019.	Nigeria		✓		Goat, sheep	Morphology
[207]	Develop a PCR-based assay for rapid discrimination between *F. hepatica* and *F. gigantica* and subsequently to apply this technique for the species identification of adult flukes collected from domestic ruminants in Nigeria.	Nigeria			✓	Cattle, sheep	Molecular
[208]	Studied gastrointestinal helminth parasites in cattle slaughtered in abattoirs in Gwagwalada Area Council.	Nigeria		✓		Cattle	Morphology
[209]	Determine the prevalence, species diversity, patterns of infections and risk factors associated with helminth infections of cattle in Ilorin, Nigeria.	Nigeria		✓		Cattle	Morphology
[210]	Ascertain the hematological parameters and *Fasciola gigantica* prevalence in trade cattle slaughtered in Ikpa abattoir, Nsukka, Southeastern Nigeria, as well as establish the association if any, between *Fasciola gigantica* infection and breed and sex of trade cattle.	Nigeria		✓		Cattle	Morphological
[211]	Investigate phenotypic and genotypic divergence existing between the hepatic flukes, *Fasciola hepatica* and *F. gigantica*.	Nigeria		✓			Morphology, molecular
[212]	Determine the prevalence of *Fasciola* spp in cattle slaughtered for consumption in Ado Ekiti, Nigeria.	Nigeria			✓	Cattle	Morphology
[213]	Determine the prevalence of gastro-intestinal parasites among the cattle in Akure, Nigeria.	Nigeria	✓			Cattle	Morphology
[214]	Provide baseline epidemiological data by estimating the prevalence rate of fasciolosis in cattle population in the study area.	Nigeria		✓		Cattle	Morphology
[215]	Determine the prevalence and the intensity of *Fasciola gigantica* infection in slaughtered cattle in Ado municipal abattoir.	Nigeria		✓		Cattle	-

[216]	Determine the prevalence of fascioliasis among slaughtered cattle in abattoirs in Akure metropolis.	Nigeria			✓	Cattle	Morphology
[217]	Investigated the prevalence of, and herd level risk factors for, fasciolosis and other trematode infections in cattle in the Edu Local Government Area (LGA).	Nigeria		✓		Cattle	Morphology
[218]	Determine the prevalence of fasciolosis in cattle slaughtered at Ubakala slaughterhouse in Umuahia South L.G.A, Abia State.	Nigeria		✓		Cattle	Morphology
[219]	Reports the prevalence of *Fasciola*, its effects on blood and liver of cattle and goat slaughtered in Calabar.	Nigeria			✓	Cattle, goat	Morphology
[220]	Determine the prevalence of gastrointestinal helminths of slaughtered cattle in southwestern Nigeria.	Nigeria		✓		Cattle	Morphology
[221]	Provide information on the seroprevalence and the status of *F. gigantica* at slaughter and on farms in Zaria, and to determine association between *F. gigantica* infection and age, sex and breed of cattle in Zaria.	Nigeria		✓		Cattle	Coprology
[222]	Determine the prevalence of fasciolopsis infection in Eke Market abattoir, Afikpo, Nigeria.	Nigeria			✓	Cattle	Morphology
[223]	Determine the prevalence and phenotypes of *Fasciola* from slaughter cattle in Maiduguri.	Nigeria		✓		Cattle	Morphology
[224]	Determine the prevalence and intensity of fascioliasis in cattle slaughtered for human consumption in Minna metropolis, Niger State, Nigeria.	Nigeria		✓		Cattle	Morphology
[225]	Determine the prevalence of fascioliasis in cattle slaughtered in abattoirs within the three Local Government areas in Benin City, Edo State, Nigeria.	Nigeria			✓	Cattle	Morphology
[226]	Determine the prevalence of helminthes parasites affecting cattle slaughtered at the Wukari Local Government abattoir, in Taraba State North-Eastern Nigeria.	Nigeria		✓		Cattle	Morphology

[227]	Investigate the status of helminth parasitic infection in West African Dwarf goats in Umuariaga Umuahia Abia State.	Nigeria	✓			Goat	Morphology
[228]	Determine the prevalence of fascioliasis among slaughter sheep in selected abattoirs in Imo state.	Nigeria		✓		Sheep	Morphology
[229]	Bring into focus the magnitude of *Dicrocoelium* and *Fasciola* infections through post-mortem identification and counting of these worms or their eggs in the liver or gall bladders of slaughtered cattle in Zaira, Nigeria.	Nigeria		✓		Cattle	Morphology
[230]	Investigate the presence and intensity of *F. gigantica* in cattle slaughtered in Onitsha Urban and environs.	Nigeria		✓		Cattle	Morphology
[231]	Determine the prevalence of *Fasciola gigantica* and *Cysticercus bovis* and some disease conditions of public health importance in the Nsukka urban abattoir.	Nigeria		✓		Cattle	Morphology
[232]	A prevalence study of fascioliasis in cattle and goats involving retrospective analysis of monthly slaughterhouse records (between 1993 and 1997) and six months meat inspection at Nsukka Urban slaughterhouse between January and June 1998 was carried out in Enugu State.	Nigeria		✓		Cattle, goat	Morphology
[233]	Determine the prevalence of *F. gigantica* and *D. hospes* in domestic livestock slaughtered at Maiduguri, the largest urban centre in the State. Prevalence was assessed by the presence of eggs in the gall bladder.	Nigeria		✓		Cattle, sheep, goat	Morphology
[234]	Investigates the prevalence, seasonality and type of helminths infesting sheep at the ILCA station.	Nigeria	✓			Sheep	Morphology
[235]	Carried out in a rural slaughter slab in Soba, Zaria province, which obtained cattle and small ruminants from livestock owners in the Soba district, generally within I0 km of that village.	Nigeria		✓		Cattle, sheep, goat	Morphology
[236]	Examined sequence variability in a portion of the mitochondrial cytochrome c oxidase subunit 1 (pcox1) and NADH dehydrogenase subunits 4 and 5 (pnad4 and pnad5)	Niger			✓	Cattle, sheep	Molecular

	among 39 isolates of Fasciola spp., from different hosts from China, Niger, France, the United States of America, and Spain.						
[237]	Characterize *Fasciola* samples from Niger from different host animals and geographical localities by sequences of the first and second internal transcribed spacers (ITS-1 and ITS-2) of ribosomal DNA (rDNA).	Niger			✓	Cattle, sheep	Molecular
[238]	Determine the prevalence of bovine fasciolosis, assess the economic importance of bovine fasciolosis due to liver condemnation in the abattoir.	Ethiopia			✓	Cattle	Morphology
[239]	Determine the prevalence of bovine fasciolosis and estimate the magnitude of direct economic loss attributed due to liver condemnation at Lalo Municipal Abattoir.	Ethiopia			✓	Cattle	Morphology
[240]	Determining the major trematode infections from the sheep owned by smallholder farmers in Lemo Woreda and its economic loss due to liver condemnation at Hossana Town.	Ethiopia	✓			Sheep	Morphology
[241]	Determine the prevalence rate and the economic significance of bovine Fasciolosis in shambu municipality abattoir, by using ante-mortem examination (feces) and post- mortem examination of liver of each slaughtered animal.	Ethiopia			✓	Cattle	Morphology
[242]	Determine the current prevalence of bovine fasciolosis and its associated risk factors as well as financial losses due to liver condemnation.	Ethiopia			✓	Cattle	Morphology
[243]	Estimate the prevalence and economic importance of fasciolosis slaughtered at Wulnchit Municipal Abattoir.	Ethiopia			✓		Morphology
[244]	Determine the prevalence of fasciolosis and *Paramphistomum* infection in cattle slaughtered at Hirna municipal abattoir.	Ethiopia		✓		Cattle	Morphology
[245]	Determining the prevalence of assessing the magnitude of infection in different equine species, sex and age groups in Asela and Goba districts of Arsi-Bale highlands of Oromiya region, Southeastern Ethiopia.	Ethiopia			✓	Horse, donkey, mule	Morphology

[246]	Determine prevalence of bovine fasciolosis and economic significance in and around chora wereda.	Ethiopia			✓		Morphology
[247]	Determining the prevalence and economic loss of fasciolosis in small ruminants slaughtered in Addis Ababa abattoir.	Ethiopia			✓	Goat, sheep	Morphology
[248]	Assess the current on farm and abattoir prevalence of bovine fasciolosis and associated economic loss at robe municipal abattoir.	Ethiopia			✓	Cattle	Morphology
[249]	Assess the overall prevalence of the disease, evaluation of the diagnostic efficiency of direct sedimentation method and the direct economic loss of illness due to liver condemnation in the study area.	Ethiopia			✓	Cattle	Morphology
[250]	Determine the abattoir-based prevalence, to point out associated risk factors of the disease and to estimate financial loss due to liver condemnation in sheep slaughtered in Bahir Dar.	Ethiopia			✓	Sheep	Morphology
[251]	Determine the prevalence of fasciolosis and associated risk factors, the test agreement between fecal examination by sedimentation and post-mortem examination, and the direct financial loss due to liver condemnation.	Ethiopia			✓	Sheep, goat	Morphology
[252]	Determine the abattoir prevalence and direct economic loss associated with fasciolosis in cattle at Angacha municipal abattoir,<br>Ethiopia.	Ethiopia			✓	Cattle	Morphology
[253]	Determine the prevalence of small ruminant fasciolosis, associated risk factor, *Fasciola* species identification and to determine the sensitivity of sedimentation and direct smear diagnostic method over post-mortem.	Ethiopia			✓	Goats, sheep	Morphology
[254]	Determine the prevalence of bovine fasciolosis and to determine the risk factor of bovine fasciolosis in Hossana municipal abattoir.	Ethiopia			✓	Cattle	Morphology

[255]	Estimate the prevalence of *Fasciola* infection and assess the associated financial loss due to liver condemnation in cattle slaughtered at Wolaita Sodo municipal abattoir.	Ethiopia			✓	Cattle	Morphology
[256]	Assess the incidence of fasciolosis in cattle and the extent of direct economic loss due to liver disapproval and not direct carcass loss at Wolkite community abattoir, Ethiopia.	Ethiopia			✓	Cattle	Morphology
[257]	Assess prevalence and economic significance of bovine fasciolosis.	Ethiopia			✓	Cattle	Morphology
[258]	Determine the prevalence of bovine trematodes and associated risk factors in animals slaughtered at Abergelle international export abattoir.	Ethiopia			✓	-	-
[259]	Determine the prevalence of fasciolosis in cattle and to estimate the financial loss attributed to fasciolosis because of liver condemnation.	Ethiopia			✓	Cattle	Morphology
[260]	Estimating the prevalence of parasitic infections and the associated risk factors for the increased infections in sheep and goats brought from different parts of the country and slaughtered at Hashim Nur's abattoir.	Ethiopia	✓			Sheep, goat	Morphology
[261]	Determine the prevalence and the species of liver flukes in indigenous cattle, to compare the diagnostic efficiency of fecal and postmortem examination (to evaluate the sensitivity of coprological test).	Ethiopia			✓	Cattle	Morphology
[262]	Estimate the prevalence of fasciolosis from slaughtered cattle population in Bahir Dar municipal abattoir.	Ethiopia			✓	Cattle	Morphology
[263]	Determine the prevalence of bovine fasciolosis and to estimate the magnitude of direct economic loss attributed due to liver condemnation at Haramaya Municipal Abattoir.	Ethiopia			✓	Cattle	Morphology
[264]	Evaluate the specificity and sensitivity of the flotation and sedimentation tests for the diagnosis of fasciolosis and economic importance of fasciolosis using postmortem examination on a total of 500 cattle slaughtered at Wolaita Soddo municipal abattoir, southern Ethiopia.	Ethiopia			✓	Cattle	Morphology

[265]	Know the prevalence of bovine fasciolosis in and around Inchini town, to determine the liver pathology and generate base line data for future research of bovine fasciolosis on the study area.	Ethiopia			✓	Cattle	Morphology
[266]	Assess gross and histopathological lesions as well as cytological changes of the liver, gall bladder and hepatic lymph nodes associated with liver fluke infection.	Ethiopia			✓	Cattle, goat, sheep	Morphology
[267]	Investigate the prevalence of gastrointestinal parasites in pigs slaughtered at AAAE, Addis Ababa, Ethiopia.	Ethiopia	✓			Pigs	Morphology
[268]	Assess the prevalence and economic losses of bovine fasciolosis due to liver condemnation at Hawzien abattoir, Tigray region, Ethiopia.	Ethiopia			✓	Cattle	Morphology
[269]	Compare two parasitological tests and postmortem examination to detect prevalence of bovine fasciolosis in West Shewa Zone.	Ethiopia			✓	Cattle	Morphology
[270]	Determine the prevalence of fasciolosis and assessment of its direct economic impact due to both partial and total liver condemnation in slaughtered cattle of Areka municipal abattoir.	Ethiopia			✓	Cattle	Morphology, coprology
[271]	Determine the prevalence and species of *Fasciola* in sheep slaughtered at Debre-Birhan municipal abattoir, also to associate the severity of liver lesions with *fasciola* infection.	Ethiopia			✓	Sheep	Morphology
[272]	Determine the prevalence of fasciolosis in cattle slaughtered at this abattoir and to assess its economic impact.	Ethiopia			✓	Cattle	Morphology
[273]	Determine the prevalence of cattle disease in Wolaitasoddo municipal abattoir, compare the diagnostic efficiencies of fecal and postmortem examination, estimate the magnitude of direct and indirect economic loss incurred due to liver condemnation and carcass weight loss.	Ethiopia			✓	Cattle	Morphology
[274]	Assess the current on farm and abattoir prevalence of bovine fasciolosis and associated economic loss in Lemo district.	Ethiopia			✓	Cattle	Morphology

[275]	Determine the prevalence of fasciolosis and *Fasciola* species among cattle slaughtered at Dangila municipal abattoir, compare the diagnostic efficiencies of fecal versus postmortem examinations and assess direct and indirect economic losses caused by fasciolosis in the study area.	Ethiopia			✓	Cattle	Morphology
[276]	Determine the prevalence of fasciolosis and the economic loss incurred due to liver condemnation in cattle slaughtered at Debre Markos municipal abattoir.	Ethiopia			✓	Cattle	Morphology
[277]	Determine the prevalence and economic significance of fasciolosis in cattle slaughtered at Mettu town municipal abattoir, Southwest Ethiopia.	Ethiopia			✓	Cattle	Morphology
[278]	Study the prevalence of fasciolosis in cattle in and around Bahir Dar and to determine the common *Fasciola* species in the area.	Ethiopia			✓	Cattle	Morphology
[279]	Estimate the prevalence of fasciolosis amongst cattle slaughtered at Elfora abattoir, Gondar and determine the consequent direct and indirect economic losses.	Ethiopia			✓	Cattle	Morphology
[280]	Determine the prevalence of bovine fasciolosis and to assess the direct economic loss due to liver condemnation in Nekemte municipal abattoir during November 2011 to March 2012.	Ethiopia			✓	Cattle	Morphology
[281]	Assess the seasonal occurrence of fasciolosis in Southwest Ethiopia, determine the magnitude and spatial distribution of fasciolosis in ruminant species managed under extensive farming system and design strategic treatment programmes for the control of the disease in the area.	Ethiopia			✓	Cattle, sheep, goat	Morphology
[282]	Investigate prevalence of GIT parasites in extensively managed pigs and associated environmental health risks in Mekelle and urban areas of southern zone of Tigray Region, Ethiopia.	Ethiopia	✓			Pig	Morphology
[283]	Estimated the prevalence of fasciolosis in cattle brought from different parts of the country and slaughtered in Addis Ababa abattoir.	Ethiopia			✓	Cattle	Morphology

[284]	Determine the prevalence, to assess the risk factors and to determine the economic loss due to liver condemnation and carcass weight loss in cattle slaughtered at Dessie municipal abattoir.	Ethiopia			✓	Cattle	Morphology
[285]	Assess the economic losses due to Ovine fasciolosis, determine the current prevalence rate and associated risk factors in Menz Lalo Midir district.	Ethiopia	✓			Sheep	Morphology
[286]	Determining the prevalence of fasciolosis in cattle and the magnitude of direct monetary loss due to liver condemnation and indirect carcass loss at Jimma municipal abattoir, Jimma town, Ethiopia.	Ethiopia			✓	Cattle	Morphology
[287]	Determine the prevalence of the parasite and its associated risk factors in the abattoir.	Ethiopia			✓	Cattle	Morphology
[288]	Estimate the prevalence of fasciolosis, assess associated risk factors, identify *Fasciola* speciecs, estimate monetary loss due to liver condemnation and estimate sensetivity of sedimentation techinique for detection of *Fasciola* eggs.	Ethiopia			✓	Sheep, goat	Morphology
[289]	Determine the prevalence of the disease and its economic impact associated to liver condemnation in Mekelle Municipal Abattoir and compare the intensity of infection with gross liver damage and identify the commonly involved fluke species.	Ethiopia			✓	Cattle	Morphology
[290]	Determine the prevalence and economic significance of bovine fasciolosis; identify species involved and evaluate the technique used for the detection of *Fasciola* (eggs and adult parasite).	Ethiopia			✓	Cattle	Morphology
[291]	Estimate the prevalence of bovine fasciolosis among cattle slaughtered at Bishooftu Municipal Abattoir considering their origin.	Ethiopia			✓	Cattle	Morphology
[292]	Determined the prevalence and risk factors of fasciolosis in cattle at Mekelle municipality abattoir using liver inspection and faecal examination techniques.	Ethiopia			✓	Cattle	Morphology

[293]	Generate baseline data on the prevalence of gastrointestinal parasites in small ruminants of north Gondar zone, Northwest Ethiopia.	Ethiopia	✓			Sheep, goat	Morphology
[294]	Survey of abattoir epidemiology and the associated monetary loss from the diseases in slaughtered sheep and goats.	Ethiopia			✓	Sheep, goat	Morphology
[295]	Study the prevalence of bovine fasciolosis and assess direct (liver condemnation) and indirect (carcass weight) economic losses caused by fasciolosis in the study area.	Ethiopia	✓			Cattle	Morphology
[296]	Determine the prevalence and the species of liver flukes in indigenous adult cattle, to compare the diagnostic efficiency of fecal and postmortem examination and to assess the economic significance of bovine fasciolosis due to liver condemnation in the abattoir.	Ethiopia			✓	Cattle	Morphology
[297]	Determining the prevalence and the economic loss due to liver condemnation in cattle slaughtered at Adwa municipal abattoir.	Ethiopia			✓	Cattle	Morphology
[298]	Estimate the prevalence of concurrent infection of hydatidosis and fasciolosis at Mekelle Municipal abattoir of Tigray Region of Ethiopia.	Ethiopia			✓	Cattle	Morphology
[299]	Investigate the epidemiology of fasciolosis in working donkeys in Ethiopia by means of a cross-sectional coprological survey and retrospective post-mortem examination finding.	Ethiopia			✓	Donkey	Morphology
[300]	Identify the species of helminth parasites: their prevalence and relative abundance: their pathogenic effect and economic significance in working donkeys in Ethiopia.	Ethiopia			✓	Donkey	Morphology
[301]	Determine prevalence and most prevalent species of liver flukes in indigenous adult cattle, to compare diagnostic efficiency of faecal and postmortem examination and to assess the economic importance of bovine fasciolosis due to liver condemnation in the abattoir.	Ethiopia			✓	Cattle	Morphology

[302]	Determine the prevalence, abundance and seasonal incidence of nematode and trematode infections of sheep and goats slaughtered at four abattoirs located in geographically separate towns in semi-arid eastern Ethiopia.	Ethiopia			✓	Sheep, goat	Morphology
[303]	Determine the monthly prevalence rate of fasciolosis in three different age categories of local breed cattle during the dry season; assess the monthly herd *Fasciola* egg output; compute the herd egg-shedding index, and compare it with the values obtained from classical coproscopic examination; and carry out an abattoir-based survey to estimate the extent of *Fasciola* infection prevalence in adult stocks and compare the intensity of infection with the severity of liver lesions	Ethiopia			✓	Cattle	Morphology
[304]	Determine the prevalence of endoparasites of the highland sheep in Ethiopia.	Ethiopia	✓			Sheep	-
[305]	Determine the prevalence and economic importance of fasciolosis in slaughtered cattle from various parts of Kenya during the 1 O-year period from 1990-1999 as well as the relative occurrence of *F. gigantica* and *F. hepatica*.	Kenya		✓		Cattle	Morphology
[306]	Determine sites of endemic areas, the incidence of the disease and the cycle of the parasite in the definitive host	Malawi		✓		Cattle	Morphology
[307]	Investigate the epidemiology of trematode infections in cattle within highland and lowland areas of Iringa Rural District, in southern Tanzania.	Tanzania		✓		Cattle	Morphology
[308]	Determine the prevalence and financial losses of Bovine fasciolosis at SAAFI and Sumbawanga municipal abattoirs.	Tanzania		✓		Cattle	Morphology
[309]	Determining the occurrence of fasciolosis in animals brought for slaughter at Kasulu district abattoir.	Tanzania		✓		Cattle, goat	Morphology
[310]	Reports results of a survey of various endoparasites in small scale goat keeping farms in the urban and peri-urban areas of Mwanza City.	Tanzania		✓		Goat	Morphology

[311]	Determining the prevalence and economic importance of liver fasciolosis in cattle slaughtered at Arusha abattoir in Tanzania.	Tanzania		✓		Cattle	Morphology
[312]	Determine the occurrence of liver fasciolosis and stilesiosis in cattle, sheep and goats slaughtered at Arusha municipal abattoir over a period of three years between 2005 and 2007.	Tanzania		✓		Cattle, sheep, goat	Morphology
[313]	Determine the prevalence of helminth infections in three cattle management systems in Iringa district, Southern Highlands of Tanzania.	Tanzania		✓		Cattle	Morphology
[314]	Determine the epidemiology of *F. gigantica* and amphistomes of cattle in three management systems in Iringa district.	Tanzania		✓		Cattle	Morphology
[315]	Identified parasites found in the livers of slaughtered cattle on the island of Pemba, Tanzania.	Tanzania		✓		Cattle	Morphology
[316]	Presents the genomes of *Fp. buski* and *Fa. gigantica*, making the genomes of all three human-infecting fasciolid flukes available and via comparisons provide a better understanding of their evolutionary history and diversification, and the genetic bases underlying their phenotypic and ecological divergence and adaptation to different host species and habitats.	Uganda		✓		Cattle	Molecular
[317]	Determine the prevalence of bovine fasciolosis and its economic impact in Kampala City abattoir.	Uganda			✓	Cattle	Morphology

Table S2. The distribution and occurrence of *Fasciola* species in Africa based on studies conducted from 1980 – 2022.

Subregion	Country	*Fasciola hepatica*	*Fasciola gigantica*	Both	Aspermic *Fasciola* sp./suspected parthenogenetic *Fasciola*/*Fasciola* hybrids	References
North Africa	Algeria	11	1	-	-	[36,37,110,140–147]
	Egypt	5	6	14	7	[1,26,29,33,38,106–109,123,148–164]
	Morocco	1	-	-	-	[165]
	South Sudan	-	1	-	-	[166]
	Sudan	-	3	-	-	[167–168]
	Tunisia	7	-	-	-	[51–53,142,147,170]
Middle Africa	Cameroon	-	2	-	-	[171,172]
	Chad	-	2	-	1	[2,173]
	DR Congo	-	1	-	-	[174]
Southern Africa	Botswana	-	2	-	-	[175,176]
	South Africa	2	1	3	1	[23,32,177–180]
	Swaziland	-	1	-	-	[181]
	Zambia	-	8	1	-	[78,151,182–189]
	Zimbabwe	1	3	1	1	[28,79,80,176,190–192]
West Africa	Burkina faso	-	2	-	-	[193,194]
	Côte d'ivoire	-	2	-	-	[195,196]
	Ghana	1	3	-	-	[166,197–199]
	Mali	-	1	-	-	[200]

	Mauritania	-	1	-	-	[201]
	Nigeria	3	25	7	-	[151,202–236]
	Niger	-	-	2	-	[237]
East Africa	Ethiopia	8	1	59	-	[5,238–304]
	Kenya	-	5	-	-	[95,96,98,99,305]
	Malawi	-	1	-	-	[306]
	Tanzania	-	10	2	-	[102,103,158,307–315]
	Uganda	-	2	1	-	[100,316,317]

Key: - no evidence of species occurrence.

Table S3. Summary of African studies reporting on the occurrence of *Fasciola* species in their snail intermediate hosts.

Author	Aim/ objectives	Country	Snail species	Species of infection	Detection method
[23]	Confirm whether *L.* (*P.*) columella was transmitting *F. gigantica* and/or *F. hepatica* in selected locations of KwaZulu-Natal and Eastern Cape provinces of South Africa.	South Africa	*Pseudosuccinea columella*	*F. gigantica, Fasciola sp.*	Molecular
[26]	Investigated the trematode species occurring in the invasive snail *P. columella*, collected from irrigation channels in the Fayoum governorate where cases of fascioliasis are commonly reported.	Egypt	*Pseudosuccinea columella*	*F. gigantica*	Molecular
[28]	Assessed the prevalence of *Fasciola* sp. infections in the gastropod populations.	Zimbabwe	*Pseudosuccinea columella*	*Fasciola sp.*	Molecular
[36]	Set the prevalence of *F. hepatica* in *G. truncatula* based on a molecular basis using multiplex PCR for the first time in Algeria.	Algeria	*Galba truncatula*	*F. hepatica*	Molecular
[37]	Determine the seroprevalence of natural fasciolosis in cattle and sheep.	Algeria	*Galba truncatula*	*F. hepatica*	-
[38]	Investigate *Fasciola* species in cattle and lymnaeid snails in Dakhla Oasis, El-Wadi El-Gadid, Egypt. Gross and microscopic findings were confirmed by duplex PCR and sequence analysis of the resulting mitochondrial cox1 amplicons.	Egypt	*Galba truncatula*	*F. hepatica*	Morphology, molecular

[40]	Identify *Fasciola* spp. infection in cattle and investigate its molluscan intermediate host with mitochondrial DNA-targeting PCR assay in Dakhla Oasis, El wadi Elgadid, Egypt.	Egypt	*Galba truncatula*	*F. hepatica*	-
[48]	Reports *Biamphalaria alexandrina* natural infection with *F. gigantica* in Egypt.	Egypt	*Biamphalaria alexandrina*	*F. gigantica*	Molecular
[51]	Study the definitive host infection as well as to identify the plants causing its contamination then at a later stage to determine the snail habitat ecological parameters, the density, the annual generation number of the intermediate host *B. truncatus* and the prevalence of its natural infection.	Tunisia	*Bulinus truncatus*	*F. hepatica*	Morphology
[52]	Report natural infection of *B. truncatus* with *F. hepatica* in the region of Sejnane (North Tunisia), as this district is known to be a zone of endemic fasciolosis.	Tunisia	*Bulinus truncatus*	*F. hepatica*	Morphology
[53]	Determine the epidemiology of fasciolasis in Gafsa oases.	Tunisia	*Galba truncatula*	*F. hepatica*	Morphology
[95]	Investigates the diversity of the digenean trematode fauna of snails from the Kenyan part of Lake Victoria.	Kenya	*Radix natalensis*	*F. gigantica*	Morphology, molecular
[96]	Determine the distribution and prevalence of infection of snails per agro-ecological zone and environmental factors in vector snail habitats.	Kenya	*L. natalensis, Biomphalaria pfeifferi, Biomphalaria sudanica*	*F. gigantica*	Morphology
[98]	Observations were made on a ranch in the Central Rift Valley of Kenya where fasciolosis was endemic.	Kenya	*Radix natalensis*	*F. gigantica*	Morphology
[99]	Studied the monthly incidence of *F. gigantica* intramolluscan stages in *L. natalensis* from endemic sites in Nyeri district, Kenya.	Kenya	*Radix natalensis*	*F. gigantica*	-
[103]	Answer the following questions. What are the intermediate hosts for *Fasciola* spp. at different altitudes? Is *F. hepatica* found at high altitude in East Africa? Is there any evidence of *F. hepatica*/*F. gigantica* hybridization in our samples?	Tanzania	*Lymnaea truncatula*, *Lymnaea natalensis*	*F. hepatica*, *F. gigantica*	Morphology, molecular

Table S4. Summary of studies reporting on the occurrence of intermediate hosts of *Fasciola* spp. in Africa.

Author	Aim/ Objective	Country	Species
[16]	Examine several African populations using combination of mitochondrial and nuclear DNA markers to determine the identity and biogeographical affinities, reconstruct the colonization history including the likely mode of dispersal, and test for the presence of cryptic *Galba* species in Africa.	Morocco, Kenya, South Africa, Lesotho, Tanzania, Ethiopia, Uganda	*Galba truncatula, Radix natalensis, Pseudosuccinea columella*, *G. mweruensis*.
[23]	To determine the infection status of *Pseudosuccinea columella* with *F. hepatica* and/or *F. gigantica* using molecular techniques.	South Africa	*Pseudosuccinea columella*
[26]	Investigated snails collected in irrigation canals in Fayoum governorate in Egypt for prevalence of trematodes with focus on *P. columella* and its role for the transmission of *F. gigantica*.	Egypt	*Pseudosuccinea columella*
[39]	Characterise both *Pseudosuccinea columella* and *Radix natalensis* using molecular analysis and shell measurements.	Egypt	*Pseudosuccinea columella, Radix natalensis*
[41]	Survey the benthic mollusc communities in River Nile and its branches in Assiut governorate, Egypt.	Egypt	*Radix natalensis, Pseudosuccinea columella*
[42]	Document the current density of the malacological fauna of Dakahlia, especially the lymnaeid snails.	Egypt	*Radix natalensis, Galba truncatula, Pseudosuccinea columella*
[43]	Determined if *Radix natalensis* might act as an intermediate host in the life cycle of *F. hepatica* in Egypt.	Egypt	*Radix natalensis*
[44]	Study the freshwater snail and larval trematode communities in Al-Salam canal and the effect of its mixed water on these communities. Study the possible establishment of snail vectors of parasitic diseases.	Egypt	*Radix natalensis*
[45]	Survey the different species of *Lymnaea* and *Biomphalaria*, for identification, map-distribution and to be sought for natural infection with the immature stages of *Fasciola* species.	Egypt	*Radix natalensis, Galba truncatula, Pseudosuccinea columella*
[46]	Studied the possible establishment of snail vectors of diseases in new settlement areas in Sinai and the seasonal abundance of these snails in irrigation and drainage channels.	Egypt	*Galba truncatula, Pseudosuccinea columella*
[47]	Document the abundance of both two *Lymnaea* species, and their infection rates with *F. gigantica* and other parasites in Giza during March 1997 – February 1999.	Egypt	*Radix natalensis, Galba truncatula, Pseudosuccinea columella*

[49]	Documents the recent status of the mollusc fauna of Birket Qarun (Fayum).	Egypt	*Galba truncatula, Radix natalensis*
[50]	Observed the seasonal transmission of *Fasciola hepatica* in sentinel sheep and the dynamics of the snail intermediate host.	Morocco	*Galba truncatula*
[54]	Inventory freshwater snail species, (2) to study their spatiotemporal dynamic, (3) to perform a spatial frame analysis of environmental parameters that might structure their spatial and seasonal trends and (4) to discuss the ecological requirement conditions for the distribution of snails' community.	Cameroon	*Radix natalensis, Pseudosuccinea columella*
[55]	To identify populations of Lymnaeidae snails collected from selected areas of the Okavango delta (OKD) in Botswana, and KwaZulu-Natal (KZN) and Mpumalanga (MP) provinces of South Africa using molecular techniques.	Botswana, South Africa	*Radix auricularia, Radix natalensis, Pseudosuccinea columella*
[56]	Provided an opportunity to assess the species richness of selected components of the aquatic macro-invertebrate fauna using semi-quantitative sampling techniques.	Botswana	*Radix natalensis*
[57]	Reports the geographical distribution and habitats of *L. truncatula* as reflected by the 723 samples on record in the NFSC.	South Africa	*Galba truncatula*
[58]	Investigated the spatial and temporal changes in the community structure of macroinvertebrate traits along an agricultural disturbance gradient in an Afrotropical River system.	South Africa	*Pseudosuccinea columella, Radix natalensis*
[59]	To assess whether it is possible to distinguish between macroinvertebrate responses (i.e. changes in macroinvertebrate community structures) towards different stressors within this multi-stressor environment. To compare the diversity-based and trait-based approaches in terms of bioindication of multiple environmental stressors.	South Africa	*Pseudosuccinea columella, Radix natalensis*
[61]	To provide an analysis of the genetic composition of larval digenean trematodes using polymerase chain reaction (PCR) and sequence analysis.	South Africa	*Radix natalensis*
[62]	Evaluated the distribution of genetic diversity at the largest geographic scale analysed to date in *Pseudosuccinea columella* by	South Africa	*Pseudosuccinea columella*

	surveying 80 populations collected during 16 years from 14 countries, using eight nuclear microsatellites and two mitochondrial genes.		
[63]	To survey Marico and crocodile rivers in order to establish the current status of the mollusc diversity for conservation purposes.	South Africa	*Galba truncatula, Radix natalensis, Pseudosuccinea columella*
[64]	Reports two Asian freshwater gastropods, *Radix rubiginosa* (Michelin, 1831) (Lymnaeidae) and *Gyraulus chinensis* (Dunker, 1848) (Planorbidae) from a facility supplying fish and plants to the aquarium trade in KwaZulu-Natal, South Africa.	South Africa	*Radix natalensis*
[65]	To assess the status of the mollusc diversity in the MooiRiver and to compare the results to the study done in 1963.	South Africa	*Radix natalensis*
[66]	To provide a comprehensive review of the diversity of gastropod molluscs in the St Lucia estuarine lake.	South Africa	*Radix natalensis*
[67]	Conducted four surveys of the molluscs in Olifants River and selected tributaries during two consecutive years at three localities situated on the Highveld and four localities situated in the Lowveld respectively.	South Africa	*Radix natalensis*
[68]	To gather morphological information on cercarial types, which, in the future, may assist in establishing possible linkages with strigea metacercarial stages in local fish populations from these areas.	South Africa	*Radix natalensis*
[69]	To determine the gastropod community structure influenced by a small number of key environmental factors; to determine the longer-term effects of environmental change on *T. granifera* within a variable estuarine setting.	South Africa	*Radix natalensis*
[70]	To provide an account of the distribution and habitats of the three *Lymnaea* species currently on record in the National Freshwater Snail Collection (NFSC) of South Africa.	South Africa	*Pseudosuccinea columella*
[71]	Examine different cercariae shed by *L. natalensis* in dams surrounding Pretoria, Gauteng Province, as well as to gather information on other life stages within the life cycles of these parasites.	South Africa	*Radix natalensis*
[72]	To determine the current status of freshwater molluscs in the park and to compare this with a survey done in 2001	South Africa	*Radix natalensis*

[73]	Evaluate the effect of the availability of more permanent water habitats on the freshwater mollusc diversity, and to compare results with the 1995 survey.	South Africa	*Radix natalensis, Pseudosuccinea columella*
[74]	Focuses on the geographical distribution and the habitats of *Lymnaea natalensis*, the snail intermediate host of the liver fluke, *Fasciola gigantica*, as reflected by the collection sites of its 4 552 samples currently on record in the National Freshwater Snail Collection (NFSC) of South Africa.	South Africa	*Radix natalensis*
[75]	Discusses species diversity and numbers of molluscs present in Kruger National Park.	South Africa	*Radix natalensis, Pseudosuccinea columella*
[76]	To document the repertoire and efficacy of avoidance responses of four snail species against leech attack.	South Africa	*Radix natalensis*
[77]	Investigate the helminthological status of captive impala (*Aepyceros melampus*).	Zambia	*Radix natalensis*
[78]	Studied the availability of freshwater snails and their infection rates with larval trematodes in the Kafue wetlands and to assess the level of trematode infections in cattle accessing the snail-infested areas during a period of highest water contact.	Zambia	*Radix natalensis*
[79]	Document and identify trematode parasites of the common hippopotamus (*Hippopotamus amphibius*) in artificial water systems of Zimbabwe.	Zimbabwe	*Pseudosuccinea columella*
[80]	Determined the epidemiology of *F. gigantica* in cattle in the highveld and lowveld communal grazing areas of Zimbabwe, and to use this information to recommend appropriate measures to control the parasite.	Zimbabwe	*Radix natalensis*
[81]	Determine experimentally the susceptibility of the common gastropod snails found in Zimbabwe to an amphistome, *C. microbothrium*, and to further determine the influence of *C. microbothrium* infection on the susceptibility of *B. tropicus* to *S. haematobium* and *S. Mattheei*.	Zimbabwe	*Radix natalensis*
[82]	Ascertain general freshwater snail diversity and specific diversity of the schistosome host snails.	Benin	*Radix natalensis*

[83]	Conduct a large-scale freshwater snail survey in Benin to assess the malacological diversity and the larval trematode infections with a focus on *Schistosoma* genus.	Benin	*Radix natalensis*
[84]	Conduct a large-scale freshwater snail survey in Katiola to assess the malacological diversity and the larval trematode infections.	Ivory Coast	*Radix natalensis*
[85]	*Schistosoma* spp. transmission sites and to determine the intermediate snail hosts (particularly *Bulinus* spp) at these sites and the parameters that influence intermediate snail host abundance and disease transmission potential.	Niger	*Radix natalensis*
[86]	Studied the land use/landcover change, physico-chemical parameters of water bodies and to understand the interplay between them and freshwater snails in an environment where a new industrial plant was established.	Nigeria	*Radix natalensis*
[87]	Determine some aspects of the geomorphology and limnology of the water bodies.	Nigeria	*Radix natalensis*
[88]	Evaluate MALDI-TOF MS profiling for the identification of both frozen and ethanol stored snail species using protein extracts from different body parts.	Senegal	*Radix natalensis*
[89]	Investigated how environmental and biotic factors influence the occurrence and abundance of the snail intermediate hosts in Ethiopian Rift Valley region.	Ethiopia	*Lymnaea natalensis, Lymnaea truncatula*
[90]	Identified factors affecting the occurrence and abundance of medically important freshwater snail intermediate hosts in the Omo-Gibe River basin, Ethiopia.	Ethiopia	*Radix natalensis, Galba truncatula*
[91]	Investigate the molluscicidal property of this plant.	Ethiopia	*Radix natalensis*
[92]	Focused on some aspects of the biology of *Lymnaea truncatula* and production of metacercariae of *Fasciola hepatica* under laboratory conditions.	Ethiopia	*Galba truncatula*
[93]	Characterized a range of populations of *Lymnaea natalensis* (Krauss, 1848) on Madagascar.	Madagascar	*Radix natalensis*
[94]	Answered this question by reporting results of an experimental infection of *L. natalensis* by *F. gigantica* under laboratory conditions.	Madagascar	*Radix natalensis*

[97]	Investigated the presence, and abundance of schistosomiasis and fascioliasis host snails and their relation to the vegetation types along the Mara River.	Kenya & Tanzania	*Radix natalensis*
[100]	Investigated the occurrence of fasciolosis in bovids at low and high-altitude areas on the slopes of Mount Elgon and assessed animal condition (i.e. body signalment).	Uganda	*Radix natalensis, Galba truncatula*
[101]	Produced habitat suitability maps for snail species known to act as intermediate hosts for parasites in Uganda, and (ii) to identify suitable/unsuitable areas of disease transmission overlapping these host snail distribution ranges, based on knowledge of optimal temperature regimes for parasitic development in the snail.	Uganda	*Radix natalensis*
[102]	Investigated the epidemiology of trematode infections in cattle under different cattle management systems in order to generate base line data that will provide information for the design of effective control strategies.	Tanzania	*Radix natalensis,*
[104]	Assessed the density and microhabitat preferences of *B. pfeifferi* in a natural habitat which was a residual pool of a river.	Tanzania	*Radix natalensis*
